# Genome-wide analyses of Shavenbaby target genes reveals distinct features of enhancer organization

**DOI:** 10.1186/gb-2013-14-8-r86

**Published:** 2013-08-23

**Authors:** Delphine Menoret, Marc Santolini, Isabelle Fernandes, Rebecca Spokony, Jennifer Zanet, Ignacio Gonzalez, Yvan Latapie, Pierre Ferrer, Hervé Rouault, Kevin P White, Philippe Besse, Vincent Hakim, Stein Aerts, Francois Payre, Serge Plaza

**Affiliations:** 1 Centre de Biologie du Développement, Université de Toulouse, 118 route de Narbonne, Toulouse, F-31062, France; 2 CNRS UMR5547, 118 route de Narbonne, Toulouse, F-31062, France; 3 Laboratoire de Physique Statistique, CNRS, Université Pierre & Marie Curie, Université Denis Diderot, ENS, 24, rue Lhomond, F-75231 Paris, France; 4 Current address: Department of Biology, McGill University, 845 Sherbrooke St W, Montreal, QC H3A 0G4, Canada; 5 Institute for Genomics and Systems Biology, Department of Human Genetics, The University of Chicago, 57th St Room, 10100 Chicago, IL 60637, USA; 6 Université de Toulouse, INSA, 118 route de Narbonne, Toulouse, F-31062, France; 7 Institut de Mathématiques, CNRS UMR5219, 118 route de Narbonne, Toulouse, F-31062, France; 8 Current address: Howard Hughes Medical Institute, Janelia Farm Research Campus, 19700 Helix Dr., Ashburn, VA 20147, USA; 9 Laboratory of Computational Biology, Center for Human Genetics KU Leuven, O&N I Herestraat 49 - 3000 Leuven, Belgium

## Abstract

**Background:**

Developmental programs are implemented by regulatory interactions between Transcription Factors (TFs) and their target genes, which remain poorly understood. While recent studies have focused on regulatory cascades of TFs that govern early development, little is known about how the ultimate effectors of cell differentiation are selected and controlled. We addressed this question during late *Drosophila *embryogenesis, when the finely tuned expression of the TF Ovo/Shavenbaby (Svb) triggers the morphological differentiation of epidermal trichomes.

**Results:**

We defined a sizeable set of genes downstream of Svb and used *in vivo *assays to delineate 14 enhancers driving their specific expression in trichome cells. Coupling computational modeling to functional dissection, we investigated the regulatory logic of these enhancers. Extending the repertoire of epidermal effectors using genome-wide approaches showed that the regulatory models learned from this first sample are representative of the whole set of trichome enhancers. These enhancers harbor remarkable features with respect to their functional architectures, including a weak or non-existent clustering of Svb binding sites. The *in vivo *function of each site relies on its intimate context, notably the flanking nucleotides. Two additional *cis*-regulatory motifs, present in a broad diversity of composition and positioning among trichome enhancers, critically contribute to enhancer activity.

**Conclusions:**

Our results show that Svb directly regulates a large set of terminal effectors of the remodeling of epidermal cells. Further, these data reveal that trichome formation is underpinned by unexpectedly diverse modes of regulation, providing fresh insights into the functional architecture of enhancers governing a terminal differentiation program.

## Background

Many studies have established that transcriptional networks control development, through determining specific programs of genome expression [1]. These gene regulatory networks (GRNs) are implemented by transcription factors (TFs) that bind to regulatory DNA sequences, known as enhancers or *cis*-regulatory modules (CRMs), to control the transcription of nearby genes. Although recruited to target genes via their DNA binding properties [2], TFs recognize only short and often degenerate motifs (reviewed in [3,4]). Consequently, thousands of putative binding sites (BSs) are scattered throughout the genome, hampering efficient prediction of CRMs [3,5,6]. The fine structure of enhancers as well as putative general rule(s) underlying their organization remain, however, poorly understood.

Although animals encode hundreds of TFs, only a few of them have been studied in detail to elucidate the regulatory logic of their target enhancers [7,8]. In *Drosophila*, current knowledge of enhancer structure mainly comes from works on early development - for example, TFs controlling segmentation and mesoderm specification [9-12]. Within these early acting networks, several studies have shown that the local enrichment for BSs (homotypic or heterotypic clustering) in evolutionarily conserved regions is a general signature of active enhancers [13-15]. Functionally related enhancers (driving similar expression patterns) often share a combination or code of *cis*-regulatory motifs, together defining a specific program of expression [11,16-18]. Whether enhancers rely on a constrained organization of *cis*-regulatory motifs or can accommodate flexibility in their number, composition and positioning is still debated (reviewed in [4,19,20]). While several studies have shown that regulatory codes are efficient to predict expression pattern [9,11,16], recent large-scale work suggests that developmental enhancers may have a more flexible architecture [10,20]. However, in-depth analyses of individual enhancers [21-24] have revealed an unexpected level of functional constraint in their intimate architecture. It has been proposed that constrained enhancers could be critical when TFs display limiting concentrations [25] - for example, to accurately integrate gradients [26]. On the other hand, enhancers that do not hold integrative properties might be of simpler architecture [27,28]. Distinguishing between these possibilities thus requires detailed analyses of the structure and regulatory logic of CRM-TF interactions that occur at late developmental stages.

Here, we focus on a GRN that controls cell morphogenesis during terminal differentiation of the *Drosophila *embryonic epidermis. The subset of epidermal cells that express the TF Ovo/Shavenbaby (Svb) [29] undergo localized changes in cell shape leading to the formation of dorsal hairs and ventral denticles, collectively referred to as trichomes [30]. Svb triggers the expression of various classes of cellular effectors in trichome cells. Developmental and genetic analyses have established that trichome formation relies on their collective action, acting together as a developmental module to promote cell shape reorganization [31-33]. The mechanisms underlying the co-expression of Svb-regulated genes in trichome cells remained yet poorly understood. A first level of regulation resides in the activity of Svb itself, which is controlled in a post-translational manner in response to small peptides encoded by the gene *polished-rice *(*pri*) [34]. Pri peptides trigger amino-terminal truncation of the Svb protein, switching its activity from a full-length repressor to a cleaved activator [34], therefore providing temporal control to the program of trichome formation [32]. However, little is known concerning how this TF recognizes and selects its target genes. Besides definition of DNA-binding specificity *in vitro *[35] and the identification of a few targets regulated by Ovo germline-specific isoforms [35,36], only a single epidermal enhancer dependent on Svb has been identified so far [31]. Thus, whether or not Svb targets genes that are co-expressed in trichome cells and have similar *cis*-regulatory elements remained an open question.

To address this question, we designed a set of computational modeling coupled to experimental approaches to identify and investigate the *cis*-regulatory logic of Svb-dependent enhancers. By systematic *in vivo *assays, we first identified a robust set of Svb target effectors, specifically expressed in trichome cells at the time of their morphological differentiation. We then searched for and identified 14 Svb-dependent epidermal enhancers driving their expression in trichome cells and investigated their functional organization. Computational analyses and experimental dissection led to a refinement of the Svb BSs bound *in vivo *and the identification of two additional motifs required for enhancer activity. Our studies further reveal that the distribution of these *cis*-regulatory motifs does not follow a stereotypical organization. Coupled to chromatin immunoprecipitation (ChIP)-seq and microarray profiling, the models built from these fine scale experiments allow efficient genome-wide identification of new enhancers that drive the specific expression of trichome effectors. In summary, our results show that enhancers driving co-expression in cells of a late GRN have variable composition and respective organization of *cis*-regulatory motifs, extending the idea that co-expressed developmental enhancers can have diverse *cis*-regulatory architectures [11,37], including for those mediating terminal stages of cell differentiation.

## Results

### Enrichment of conserved binding sites in Svb downstream genes

**Figure 1:**
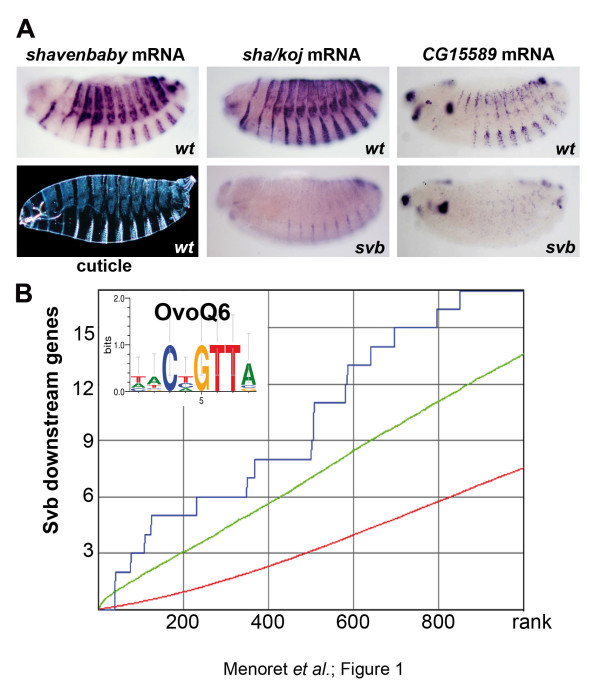
**Enrichment in binding sites defines an evolutionarily conserved signature of *svb *downstream genes**. **(a) **Expression of *svb *mRNA determines the epidermal cells that form trichomes, visible on the larval cuticle. *In situ *hybridization shows mRNA expression of two *svb *downstream genes, *shavenoid *(*sha/koj*) and *CG15589*, in wild-type (wt; top) and *svb *mutant embryos (bottom). **(b) **Receiver operating characteristic curve showing significant enrichment in putative Svb binding sites (OvoQ6 position weight matrix) among the 39 Svb downstream genes (y-axis) compared to a randomized set of 1,000 *Drosophila *genes (x-axis) using cisTargetX. The blue curve shows the detection of Svb downstream genes, the red curve a random distribution, and the green curve shows a 2 sigma interval from random.

Previous work has identified a dozen genes activated by Svb, each contributing to epidermal cell remodeling [31,33,38,39]. To investigate the *cis*-regulatory logic of Svb-dependent targets, we first sought to define a larger set of Svb downstream genes appropriate for *in silico *analyses. We therefore analyzed additional candidates selected because of their expression in subsets of epidermal cells (from the Berkeley *Drosophila *Genome Project) using *in situ *hybridization. Of 57 candidates, we identified 21 Svb-dependent genes, that is, those downregulated in *svb *mutants and upregulated following *svb *ectopic expression (Figure 1a; Figure S1A in Additional file 1 (legend in Additional file 2)), while the other 36 epidermal genes were found to be independent of Svb (Additional file 1, Figure S1B). Together with genes identified previously [31,33,38,39], this constitutes a robust set of 39 genes activated by Svb to be expressed in trichome cells. We used these 39 Svb targets to examine whether they display an evolutionarily conserved signature in their non-coding regions when compared with all *Drosophila *genes, or the 36 epidermal genes independent of *svb *as a negative control. cisTargetX aims at detecting motifs enriched among a group of co-expressed genes - for example, to predict direct targets of a TF [40]. It exploits a library of >3,000 motifs, including TF BSs and ultra-conserved DNA words [41], each motif being ranked with a score representative of both clustering and evolutionary conservation [40]. When applied to Svb targets, four of the top five motifs match the consensus CnGTT (Figure 1b; Figure S1C in Additional file 1), characteristic of the Ovo/Svb BS CnGTTa as defined *in vitro *[35]. From the 39 input genes, CisTargetX determined an optimal subset of 16 Svb direct targets, having the highest scores for the OvoQ6 motif (Figure 1b; Figure S1C in Additional file 1) [35,36]. OvoQ6 was specific to Svb targets since it was not detected in control epidermal genes (Figure S1C in Additional file 1). In contrast, motifs matching the BS of TFs involved in general epidermis differentiation, such as Grainy head [42] or Vrille/c-EBP [43], were highly ranked in Svb-independent genes (Figure S1C in Additional file 1) while lowly ranked in Svb downstream genes. Hence, OvoQ6 motifs appear to be a signature of a subset of genes activated by Svb, a result consistent with their direct regulation.

### Distribution of Svb binding site clusters poorly correlates with enhancer activity

**Figure 2:**
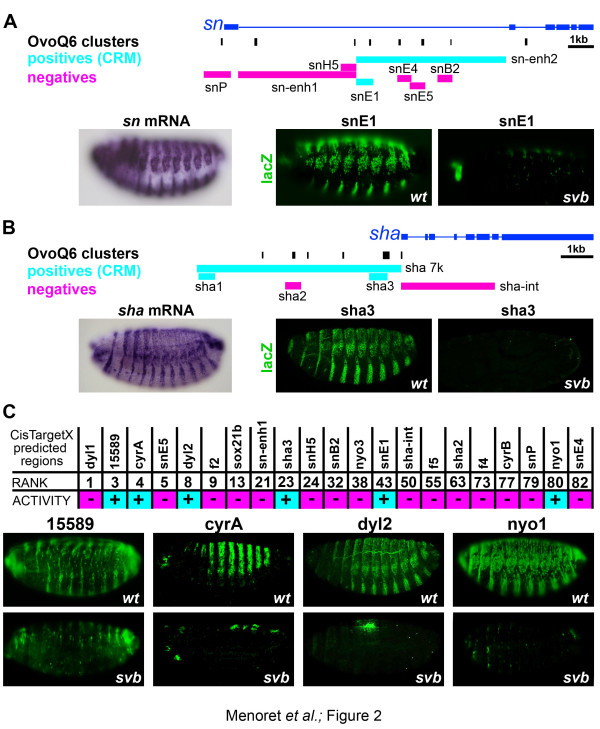
**A subset of Svb binding sites corresponding to functional enhancers**. Svb-dependent trichome enhancers were identified by transgenic reporter gene assays, from a systematic scanning of the **(a) ***singed *(*sn*) and **(b) ***shavenoid *(*sha*) genes and **(c) **regions predicted by cisTargetX. (a,b) Vertical black lines represent evolutionarily conserved OvoQ6 clusters (at least two motifs in a 1 kb window), as predicted by cisTargetX. Horizontal boxes summarize regions tested by transgenic assays, using immunostaining of the lacZ reporter (green). Negative regions (pink) do not drive specific expression in the embryonic epidermis. Regions in cyan display enhancer activity reproducing endogenous expression in trichome cells (as assayed by mRNA *in situ *hybridization, purple). The *snE1 *and *sha3 *enhancers are under the control of Svb, as demonstrated by reduced expression in *svb *mutants. (c) Putative enhancers (CRMs) predicted by cisTargetX, from clustering and/or evolutionary conservation of OvoQ6 sites. Pictures show expression of positive enhancers (cyan) in wild-type (wt; top) and *svb *mutant (bottom) embryos. Additional regions (pink) showed no detectable activity during embryogenesis.

We then examined the genomic distribution of OvoQ6 motifs within Svb target loci showing significant enrichment compared to random *Drosophila *genes. We found that each target gene contained evolutionarily conserved OvoQ6 scattered throughout intergenic and intronic regions (Figure 2a, b), instead of OvoQ6 clusters enriched locally (even using relaxed conditions of at least two sites per kilobase). To delineate which regions mediate epidermal expression, we generated a series of transgenic reporters that systematically scan two Svb downstream genes. We focused on *singed *since it encodes Fascin, a conserved regulator of actin organization [44], and *shavenoid*, which encodes a pioneer protein but displays an extreme trichome phenotype upon inactivation [31]. Although most regions with OvoQ6 sites did not show embryonic expression, we identified three sequences, one in *singed *(*snE1*) and two in *shavenoid *(*sha1 *and *sha3*), that drove expression in the epidermis, specifically in trichome cells (Figure 2a, b). Unexpectedly, one of the three sequences, *sha1*, displays a single recognizable OvoQ6 motif (see below) in *D. melanogaster*, as well as in sibling species. The activity of all three regions was lost when introduced into a *svb *null mutant background, showing that they are functional Svb target enhancers (Figure 2a, b). cisTargetX predicts the location of putative enhancers within each gene [40] and two out of three enhancers defined *in vivo *matched these predictions, in one case (*sha3*) at the highest rank for this gene (Figure 2c). We therefore investigated whether evolutionarily conserved OvoQ6 sites were sufficient to predict trichome enhancers and assayed 18 additional regions (Figure 2c) taken from the top 100 predictions. Transgenic reporter assays identified four novel sequences from *CG15589*, *cypher*, *dusky-like *and *neyo *driving expression in the epidermis. We verified in each case that they were specifically expressed in all (*dyl2*, *nyo1*) or subsets (*15589*, *cyrA*) of trichome cells where Svb is active. Consistently, these four enhancers depended on Svb since they displayed a strong reduction in their expression in the absence of *svb *(Figure 2c). Hence, analysis of Svb downstream targets shows that they are enriched in OvoQ6 BSs, a feature well conserved across *Drosophila *species. However, putative trichome enhancers predicted from evolutionary conservation and clustering of OvoQ6 sites were validated at a rate of only 28% (6/21; Figure 2c), most tested regions being devoid of activity in embryos, suggesting that other criteria distinguish enhancers from negative regions.

We noticed that OvoQ6 clusters failed to predict a number of active enhancers. This was the case for *sha1 *(Figure 2) and *Emin*, an epidermal enhancer previously identified in the gene *miniature *[31]. Examination with Cluster-Buster [45] and Swan [46] did not detect supplementary OvoQ6 in *sha1 *or *Emin *sequences (even in *D. melanogaster *only), explaining why these enhancers, containing a single Svb BS, are not included in *in silico *predictions. Six additional enhancers identified during initial stages of this study using alternative prediction criteria (Figure S1C in Additional file 1) were not highly ranked by cisTargetX because they lack BS clustering and/or evolutionary conservation. These data therefore show that BS clustering is not an absolute requisite for Svb regulation (Figure 2c), suggesting that additional sites are required to discriminate between enhancers and inactive regions.

### *De novo *motif discovery identifies a specific signature of Svb binding sites active *in vivo*

**Figure 3:**
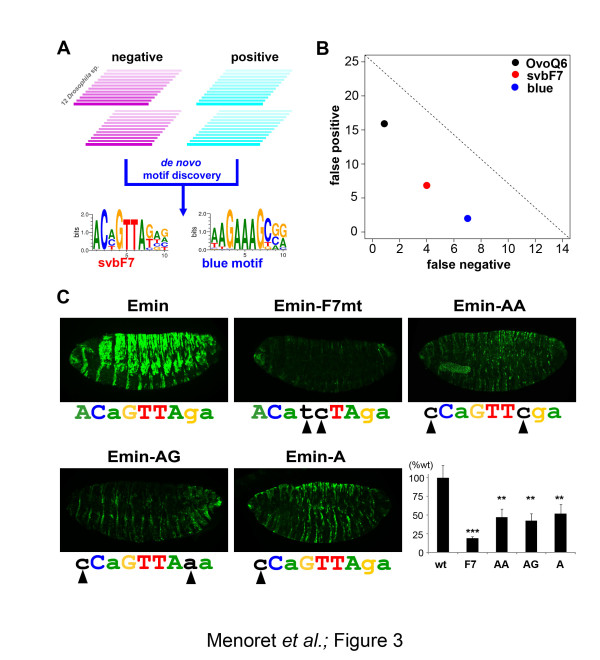
**Computational analyses allow refinement of functional Svb binding sites**. **(a) **Statistical analysis of positive enhancers versus negative regions (intergenic genomic sequences used as background) was performed for *de novo *discovery of motifs, showing evolutionary conservation across *Drosophila *species and characteristics of active enhancers. **(b) **The svbF7 and blue motifs perform best in discriminating between positive enhancers and negative regions as illustrated by the Pareto plot. **(c) **While disruption of the core CnGTT OvoQ6 motif abolished *Emin *activity, point mutations that affect the 5' and 3' flanking nucleotides strongly reduced epidermal expression, as shown by anti-lacZ immuno-staining and quantification of fluorescence signals. wt, wild type. Error bars represent the standart deviation. *** *P*-value < 0.001; ** *P*-value < 0.01.

To search for putative active Svb binding sites, we compared the two sets of experimentally tested regions - that is, the 14 enhancers (positive) and 25 inactive regions (negative) - using *Imogene*, an algorithm designed for *de novo *motif discovery [47]. Briefly, we systematically searched, *ab initio*, for 10 bp motifs that are evolutionarily conserved across Drosophilidae and display a distribution within each region statistically different from background sequences. We then evaluated how well each motif discriminated between enhancers and inactive regions and ranked these *de novo *motifs accordingly (Figure 3a). Strikingly, the most discriminative motif overlaps OvoQ6 (CnGTTa), with a similar core consensus but extending to adjacent nucleotides (*A*CHGTT*AK*). A second discriminative motif (WAGAAAGCSR), called the blue motif, was also found, and is discussed below.

The *A*CHGTT*AK *motif, hereafter called svbF7, was sufficient to detect 10 out of 14 enhancers (Figure 3b). The proportion of svbF7-positive enhancers reached 13/14, when relaxing the penalty imposed for poor conservation [47]. In contrast, svbF7 was found in only 6/25 negative regions (Figure 3b), even when lowering the threshold (data not shown). Once added to the cisTargetX library, svbF7 is the most significant motif found in the set of 39 Svb downstream genes (Figure S1C, D in Additional file 1). It also increased the accuracy of enhancer predictions, with 3 additional positives (*32159*, *Emin *and *EminB*) while 9 negatives were removed from the top 100 cisTargetX regions (Figure S1C in Additional file 1). Hence, svbF7 performs better than OvoQ6 or any other related motifs [48] (Figure 3b; Figure S1D in Additional file 1). To evaluate whether this slight extension of the Svb BS was relevant for activity, we substituted nucleotides flanking the core CnGTTa in the single svbF7 of *Emin *- that is, we altered the svbF7 motif without disrupting the OvoQ6 consensus sequence (Figure 3c). When assayed *in vivo*, different patterns of flanking substitutions, including a single point mutation of the 5' A residue, were sufficient to strongly reduce *Emin *expression (Figure 3c). This demonstrates the functional importance of flanking nucleotides within the svbF7 motif for CRM activity. Hence, our computational analysis of Svb-dependent enhancers has discovered a refined nucleotide sequence required for *in vivo *regulation.

### Trichome enhancers use different combinations of *cis*-regulatory motifs

**Figure 4:**
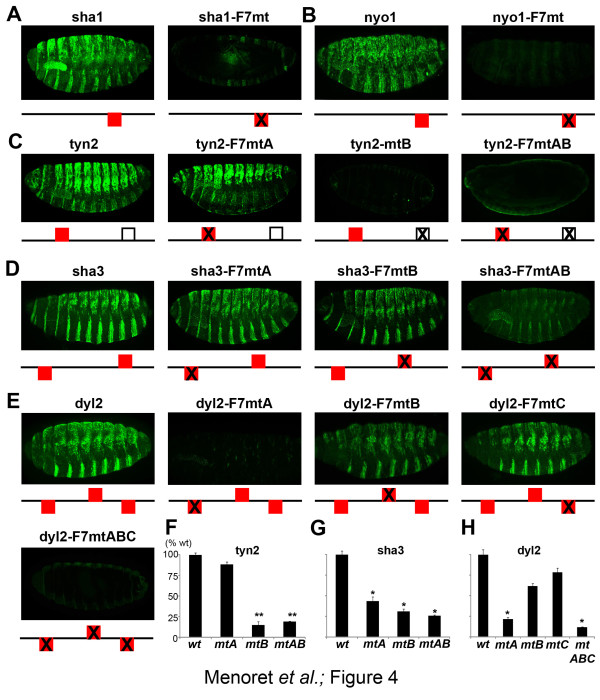
***In vivo *role of svbF7 motifs in Svb-dependent enhancers**. Anti-LacZ staining (green) shows modifications of reporter gene expression resulting from individual and simultaneous inactivation of svbF7 motifs in **(a) ***sha1*, **(b) ***nyo1*, **(c) ***tyn2*, **(d) ***sha3 *and **(e) ***dyl2 *enhancers. Red boxes schematize evolutionarily conserved svbF7 motifs; the open black box, a site that does not appear conserved across Drosophilidae. **(f-h) **Quantification of residual activity following individual disruption of svbF7 sites. **P-value < 0.01; *P-value < 0.05. wt, wild type. Error bars represent the standart deviation.

Having shown the role of svbF7 in *Emin*, we investigated its functional significance in other enhancers. We focused on enhancers containing from one to three predicted SvbF7 sites, to address the importance of single versus clustered BSs for trichome cell expression. As observed for *Emin*, disruption of the single svbF7 site abolished the activity of both *sha1 *and *nyo1 *(Figure 4a, b). The mutation of svbF7 also decreased the activity of *tyn2*, albeit weakly and only in ventral cells (Figure 4c). In this enhancer, however, we detected a second putative site that appears less conserved across species. Its inactivation strongly reduced expression (Figure 4), showing that this site mainly contributes to *tyn2 *activity. For *sha3 *and *dyl2*, which contain two and three svbF7 sites, respectively, simultaneous inactivation of these sites abrogated expression (Figure 4d, e). The individual disruption of svbF7 sites nonetheless led to varying defects. The two svbF7 sites of *sha3 *are partly redundant, their individual knockout showing similar and limited impacts when compared to their simultaneous knockout (Figure 4d-g). In contrast, a single svbF7 site plays a major role in *dyl2 *activity, whereas the two others contribute marginally to expression pattern or levels (Figure 4e, h). Hence, the disruption of svbF7 leads to reduced expression for all enhancers that have been tested, confirming the functional importance of this motif. Nevertheless, the introduction of two copies of the svbF7 motif within negative regions (*sha2 *and *12063*) was not sufficient to promote expression in trichome cells. In addition, the individual inactivation of multiple svbF7 sites has different consequences on enhancer activity, suggesting that additional elements are likely to modulate, locally, the *in vivo *function of svbF7.

**Figure 5:**
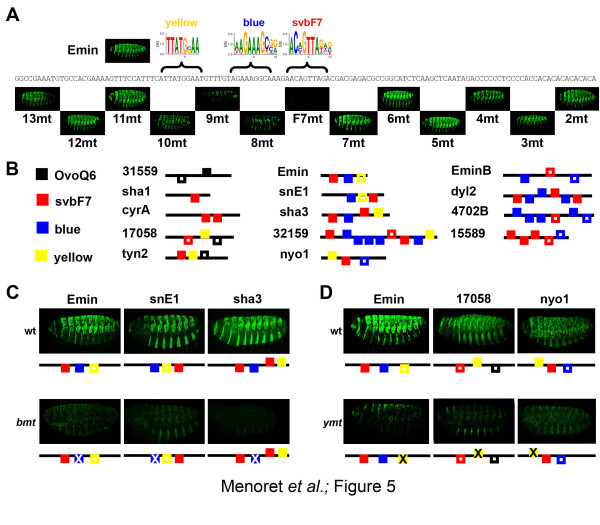
**Svb-dependent enhancers use various combinations of *cis*-regulatory elements**. **(a) **Linker-scanning mutagenesis of *Emin *identifies other 10 bp regions required for full transcriptional activity, as deduced from altered patterns of lac-Z immuno-staining (green). Positions of SvbF7, blue and yellow motifs are indicated at the top. **(b) **Black, red, blue and yellow boxes schematize the distribution, number and orientation of OvoQ6, svbF7, blue and yellow motifs, respectively. Filled boxes represent motifs conserved across *Drosophila *species, open boxes those detected only in *D. melanogaster*. **(c) **Point mutations that disrupt the blue motif in *Emin*, *snE1 *and *sha3 *reduce the activity of all three enhancers, to 40 ± 14% (*P *< 0.03), 44 ± 6% (*P *< 0.001), 8 ± 4% (*P *< 0.001) of wild-type (wt) levels, respectively. **(d) **Point mutations that disrupt the yellow motif in *Emin*, *17058 *and *nyo1 *reduce the activity of all three enhancers, to 20 ± 7% (*P *< 0.03), 16 ± 8% (*P *< 0.03), 6 ± 3% (*P *< 0.03) of wild-type levels, respectively.

We thus searched for additional *cis*-regulatory motifs and evaluated their contribution to the activity of trichome enhancers. As a first approach, we performed a systematic mutagenesis of the *Emin *enhancer by linker scanning (Figure 5a). In addition to svbF7, whose inactivation abolished *Emin *activity (F7mt), the mutation of three regions (8mt, 9mt and 10mt) strongly decreased epidermal expression, two others (3mt, 4mt) affecting only the *Emin *pattern ventrally (Figure 5a). These results show that while Svb acts as a main switch for *Emin *activity, other motifs are required for complete expression. Interestingly, our *de novo *motif discovery identified a second discriminative motif (WAGAAAGCSR), hereafter called the blue motif, enriched in positive regions and evolutionarily conserved in 7 out of 14 enhancers (Figures 3a, b and 5b). Mutations that disrupted the blue motif (9mt and 8mt) of *Emin *displayed the strongest effect, besides svbF7 knockout (Figure 5a). These unbiased data show that the blue motif represents an element that, in addition to svbF7, is critical for *Emin *activity. To further test its contribution to the activity of trichome enhancers, we mutated the blue motif in two other enhancers that contain a single occurrence of it (Figure 5b). As observed for *Emin*, disruption of the blue motif reduced *snE1 *expression (Figure 5c). Furthermore, the blue motif plays a key role in *sha3 *activity, its inactivation abolishing expression (Figure 5c), similar to the simultaneous mutation of both svbF7 sites (Figure 4d). In addition, we noticed that one important region for *Emin *expression (10mt; Figure 5a) matches an 8mer (TTATGCAA), previously predicted as a regulatory element from discovery of ultra-conserved DNA words in the genome of distant *Drosophila *species [41]. Although not sufficient by itself to discriminate between active enhancers and negative regions (data not shown), this motif, which we call the yellow motif, was nevertheless retrieved in six additional trichome enhancers (Figure 5b). To further assay *in vivo *the role of the yellow motif, we generated mutant versions of the *17058 *and *nyo1 *enhancers that disrupt their yellow motifs. As observed for *Emin*, mutation of the yellow motif led to a strong decrease in the expression driven by both *nyo1 *and *17058 *(Figure 5d), showing that the yellow motif represents a functional *cis*-regulatory element in a subset of enhancers.

Taken together, these data support that svbF7 is a main feature of Svb targets, this motif being shared by the vast majority (13/14) of active enhancers. Our analyses have discovered two additional *cis*-regulatory elements, the blue and yellow motifs, present in overlapping subsets of trichome enhancers (9/14 and 7/14, respectively). While the three motifs are present in various patterns and combinations (Figure 5B; Figure S2 in Additional file 1), functional assays demonstrated that each of them contributes to the *in vivo *activity of this sample of trichome enhancers.

### Genome-wide prediction of Shavenbaby target enhancers

**Figure 6:**
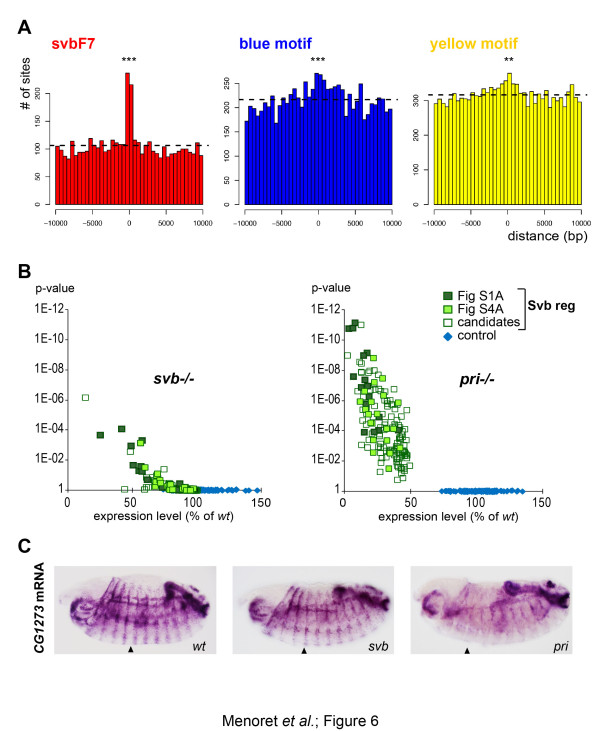
**Genome-wide profiling of embryonic genes regulated by Svb**. **(a) **Cross-correlation between conserved svbF7, blue or yellow motif instances and Svb ChIP-seq peaks throughout the whole genome. Plots show the number of motifs found in a 10 kb window on each side of the center of peaks. The *P*-value for correlation (Chi2 test) is <1E-46, <1E-9 and <1E-2 for svbF7, blue and yellow motifs, respectively. *** indicates a *P*-value < 0,001, ** < 0,01. Dashed line shows the average number of sites across the region. **(b) **Modifications in mRNA levels as measured by microarrays between wild-type (wt) and *svb *(left) or *pri *(right) embryos in Svb-regulated (green) and control (blue) sets of genes. Dark green dots represent known Svb targets (Figure S1A in Additional file 1), light green novel target genes as validated by *in situ *hybridization (Figure S4 in Additional file 1) and open dots additional candidates. **(c) **Whole mount *in situ *hybridization of *CG1273*, a Svb downstream target identified from microarray profiling, down-regulated in trichome cells (arrowheads show a raw of ventral trichome cells) of *svb *mutants and showing further reduced expression in *pri *mutant embryos.

To address whether these *cis*-regulatory motifs were a relevant signature of the genome-wide set of enhancers regulated by Svb, we undertook ChIP-seq to obtain an extensive mapping of Svb binding sites in epidermal cells. To improve specificity, we used a Svb::GFP transgene driven in ventral and dorsal trichome cells by two complementary *svb *cis-regulatory regions [34], likely at levels comparable to endogenous since it rescues *svb *mutant phenotypes [49]. ChIP-seq data indicated that Svb was bound to almost 6,000 genomic sites, a large number of binding events being a feature shared by several *Drosophila *TFs [6,8,15]. Analysis of ChIP peaks with *i*-cisTarget [50] showed that svbF7 and OvoQ6 are the most enriched motifs. A strong cross-correlation between conserved svbF7 and the center of ChIP peaks confirmed the importance of this motif (Figure 6a). As observed in our pilot analysis of enhancers, we did not detect high svbF7 clustering, multiple svbF7 motifs being rarely found within genome-wide ChIP peaks. Blue motifs (and to a lesser extent yellow motifs) also displayed a significant but weaker correlation with Svb peaks, consistent with wider genomic distribution (Figure 6a).

With the large number of Svb bound regions detected by ChIP-seq, it was unlikely that all of them were functional in the regulation of target genes [5,15]. Therefore, in order to identify the entire set of genes regulated by Svb, we performed microarray profiling, comparing wild-type to mutant embryos. In mRNA samples prepared from *svb *whole embryos, we often detected only a modest reduction in the levels of validated targets (Figure 6b; Figure S3 in Additional file 1), challenging unambiguous identification of Svb downstream genes. In the absence of *pri*, Svb behaves as a dominant repressor [34] and consistently we observed a stronger decrease in the levels of known Svb targets in *pri *mutants (Figure 6b, c; Figure S3 in Additional file 1), therefore providing an additional criterion to identify genes regulated by Svb. Henceforth, we selected the genes down-regulated in *svb *mutants and that also displayed a further (more than two-fold) reduction in their expression in *pri *mutants, as benchmarked for known Svb targets. This defined a set of 150 genes encompassing 16/39 Svb targets validated *in vivo *(Figure S1A in Additional file 1), as well as 42 additional epidermal candidates (Figure S3 in Additional file 1). Among these, we examined 23 genes by *in situ *hybridization and confirmed that 21 of them required Svb to be expressed in trichome cells (Figure 6b, c; Figure S4 in Additional file 1). These results therefore show that microarray profiling has defined a representative set of genes activated by Svb in trichome cells.

Focusing on this genomic set of Svb-regulated genes, we found 172 peaks associated with 85 genes (Figure S3 in Additional file 1), including 11 out of 14 active enhancers (Figure S7 in Additional file 1). Within the whole set of relevant Svb-bound regions, we retrieved the characteristic features of *cis*-regulatory motifs as defined previously. Although retrieved in many Svb-bound regions (Figure 6a; Figure S5 in Additional file 1), the enrichment of yellow motifs within ChIP peaks associated with Svb-regulated genes does not reliably reach a significant threshold, consistent with a broad genomic distribution [41]. In contrast, we found clear association of svbF7 motifs and to a lesser extent of blue motifs (Figure S5 in Additional file 1). Importantly, these motifs were not detected in peaks associated with a control set of genes independent of Svb (Figure S5 in Additional file 1), strongly supporting that they are hallmarks of Svb-target enhancers. As an independent way to evaluate this conclusion, we used *ab initio *analysis of ChIP peaks using PeakMotif [51]. This identified the motif ACAGTTA, which is characteristic of peaks associated with Svb downstream genes and extensively matches svbF7 (Figure S6 in Additional file 1). A second sequence (TGAAAAG), partly matching the blue motif, was also detected in about 50% of peaks, again only in Svb-regulated genes and not among control genes (Figure S6 in Additional file 1).

**Figure 7:**
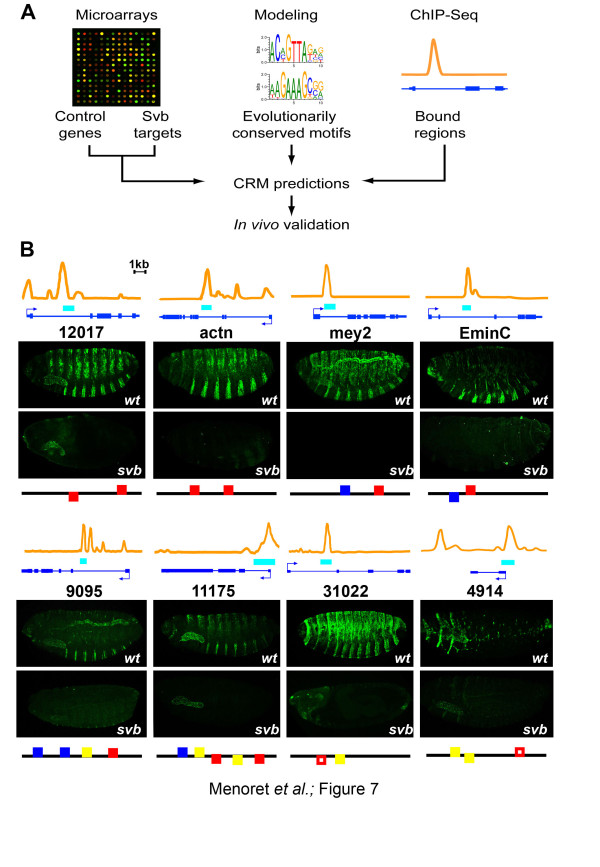
**Identification of Svb direct targets and their trichome enhancers using computational and *in vivo *experimental approaches**. **(a) **Flow diagram summarizing the pipeline used for enhancer prediction and validation. **(b) **Motif distribution coupled to ChIP-seq allows prediction of location of enhancers in Svb downstream targets. Graphs show ChIP intensity at the time of trichome formation (12 to 14 h of embryogenesis). Active enhancers are drawn as cyan rectangles. Pictures show reporter gene expression driven by corresponding regions in wild-type (wt) and *svb *mutant embryos, as revealed by anti-lacZ immunostaining (green). The composition, orientation and respective positioning of svbF7 (red), blue and yellow motifs is schematized by filled (evolutionarily conserved) and open (not traceable across species) boxes.

**Figure 8:**
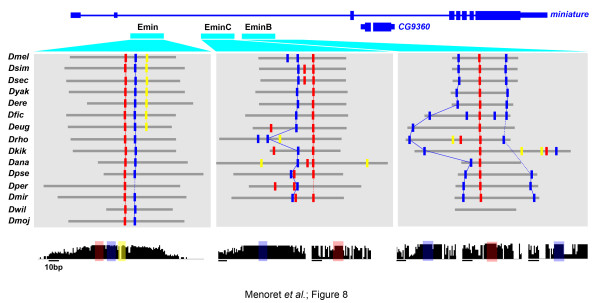
**Pattern of evolutionary conservation of the three enhancers driving *miniature *expression in trichome cells**. The position of epidermal enhancers is shown by cyan boxes and their respective architectures with respect to svbF7 (red), blue and yellow motifs are schematized across *Drosophila *species. Orthologous sequences were identified by BLAST and manually adjusted for optimized alignment. Motif search was performed in individual sequences taken independently, using the same threshold for each motif in all cases. Bottom histograms represent the pattern of evolutionary conservation across *Drosophila *species, focusing on individual regions harboring identified *cis*-regulatory motifs (color coded).

Hence, we interpret these results to imply that svbF7, and to a lesser extent the blue and/or yellow motif, would allow prediction of the location of additional trichome enhancers (Figure 7a). To evaluate this, we tested ChIPed regions containing svbF7 alone (*12017*, *14395*), svbF7 in association with either the blue motif (*mey2*, *EminC*, *actn*, *12017-2*) or the yellow motif (*31022*, *4914*), or all three motifs together (*9095*, *11175*) (Figure 7b; Figure S7 in Additional file 1). We found that 8/10 (80%) of these regions act as Svb-dependent enhancers when assayed *in vivo *(Figure 7b). Indeed, they drove robust expression, specifically in trichome cells, and their activity was reduced in *svb *mutant embryos (Figure 7b). Moreover, these data confirm that trichome enhancers are generally built from different combinations of the three *cis*-regulatory motifs. For example, only a subset of newly predicted trichome enhancers relies on the blue motif, since *mey2*, *EminC*, *9095 *and *11175 *contain conserved blue motifs whereas *12017*, *31022 *and *4914 *do not (Figure 7b; Figure S7 in Additional file 1). In the case of the *actn *enhancer, there are four partly degenerate blue motifs in the sequence from *D. melanogaster *and sibling species, while it is not retrieved in more distant species, suggesting a turnover of *cis*-regulatory motifs (Figure S8 in Additional file 1). However, aside from a couple of fast evolving enhancers, we found in many cases remarkable conservation of svbF7, blue and yellow motif patterns within individual enhancers across distantly related *Drosophila *species (Figure 8; Figure S8 in Additional file 1).

Therefore, the regulatory signatures derived from modeling and experimental dissection of a subset of enhancers helps in understanding how the Svb TF selects the genomic set of its direct targets. Furthermore, they collectively allow efficient identification of CRMs that specify the program of trichome-specific expression in response to Svb.

## Discussion

It is well established that the Shavenbaby TF determines trichome fate [29,32,52]; however, little was known on the repertoire of its direct target genes and mechanistic insights into the functional organization of trichome enhancers were lacking. Combining functional dissection, computational modeling and genome-wide profiling, we provide here a molecular map of the ultimate repertoire of genes and *cis*-regulatory elements implementing the network of trichome differentiation.

### Physical elements of the GRN governing trichome formation

Our results identify a high-confidence set of more than 150 genes activated by Svb in trichome cells. We confirmed 60 of these, showing complete or partial down-regulation in the absence of active Svb protein. While most genes are expressed in all trichome cells, some are restricted to trichome subsets, suggesting that they can contribute to the diversity of trichome shape and organization observed along the body [52]. Functional annotation (Gene Ontology and manual curation) indicates that Svb controls terminal players of trichome differentiation. In addition to novel factors of F-actin organization [31,39], extracellular matrix remodeling [31,33], cuticle formation [31,38] and pigmentation [31], we identify enzymes involved in oxidation-reduction, proteolysis and cell trafficking, further extending the repertoire of cellular functions involved in the terminal differentiation of trichome cells. Hence, a major role of Svb in trichome formation is to directly activate the expression of a battery of cell morphogenesis effectors. In support of this, ChIP-seq peaks are present in >70% of these Svb-dependent effector genes. Experimental assays further validated 22 functional enhancers driving the expression of genes encoding factors involved in cytoskeletal or extracellular matrix reorganization, sugar binding, proteolysis and additional enzymes.

Recent work has established that apparently redundant, or shadow, enhancers ensure robust expression of TFs [53,54]. For example, the transcription of *svb *itself involves separate enhancers that buffer the trichome pattern against variations in the genetic background and external conditions [53]. It has been proposed that shadow enhancers are required to drive acute expression of some key developmental regulators [55]. We define within both *shavenoid *and *miniature *separable enhancers (*sha1*, *sha3*, *Emin*, *EminB*, *EminC*) that mediate Svb regulation. These data indicate that apparently redundant enhancers may not be limited to regulatory factors operating at high hierarchic positions in gene networks. Instead, we provide evidence that several 'blue collar' effector genes display a similar regulatory architecture, suggesting that multiple enhancers represent an overlooked feature of the successive tiers of gene networks.

### Binding site clustering as a general signature of active enhancers?

Early acting enhancers often comprise multiple BSs for a given TF [56,57]. For example, conserved BS clusters have identified target enhancers of Dorsal [13] or Bicoid [58] and feature functional Twist-bound regions [15]. Of note, most algorithms developed for enhancer detection extensively use motif clustering as an important predictor [59]. We found a clear enrichment in putative Svb BSs (OvoQ6 motif) in its downstream genes; however, only a small proportion of these motifs mediate *in vivo *regulation. There is very limited, if any, clustering of Svb BSs in ChIP peaks associated with Svb target genes, and even genome-wide. Within the trichome enhancers we validated experimentally, 13 out of 22 display a single Svb site. Furthermore, for the enhancers *tyn2*, *sha3 *and *dyl2*, which contain two to three Svb BSs, the inactivation of individual sites has often limited consequences, as also reported for other TFs [60]. Even if some sites have been missed by computational approaches, the presence of multiple BSs within a short region is not a deterministic feature of active Svb-dependent enhancers.

These findings highlight a paradoxical discrepancy between the enrichment of putative BSs accumulated in Svb downstream genes and the limited number of those acting as *cis*-regulatory elements. Is there a role for this evolutionary accumulation of Svb-like motifs in Svb targets? For example, these sites with presumably weaker affinity (at least *in vivo*) can increase the local concentration of the TF facilitating regulation through a few BSs stably bound *in vivo*, as it has been suggested on thermodynamics grounds [61] or to explain the existence of thousands of binding events that are transcriptionally inactive [5,15].

### Trichome enhancers rely on diverse combinations of *cis*-regulatory motifs

We found that the motif bound by Svb *in vivo *is more constrained than the consensus defined from *in vitro *[35] or one-hybrid approaches [48]. This shows that slight sequence differences, not detected *in vitro*, can play a key role within genomic context [62], such as revealing the influence of co-factors [63].

In addition, other motifs influence which Svb BSs are functional as regulatory elements, a notion well in line with recent results on the *in vivo *specificity of Hox factors [64]. Our statistical approaches identified a more widely spread 'blue' motif. Importantly, only half of the enhancers comprise blue motifs, indicating that there are several ways to build Svb-responsive enhancers. Indeed, the systematic dissection of Emin disclosed an additional motif (TTATGCAA) ultra-conserved across Drosophilidae [41] and contributing to its activity. This 'yellow' motif is retrieved in half of the trichome enhancers, with or without blue motifs. It is, however, barely specifically enriched in Svb-bound regions and therefore was not predicted by our computational analyses (positives versus negative regions), showing the importance of unbiased functional dissection to disclose the full spectrum of *cis*-regulatory elements. Indeed, the disruption of either blue or yellow motifs strongly affects enhancer function in all tested cases, providing experimental evidence of their *cis*-regulatory activity.

Trichome enhancers thus display various combinations of motifs, from those containing only Svb BSs (5/22), Svb plus yellow (4/22), Svb plus blue (6/22) or all three together (7/22). These different motif compositions do not appear to correlate with distinct subclasses of gene function (DM, unpublished data). Furthermore, multiple enhancers from the same gene can harbor distinct combinations, as exemplified by *shavenoid *and to a lesser extent by *miniature *(Figure 8; Figure S6 in Additional file 1). Several studies have shown that motif composition may correlate with a given spatio-temporal pattern - for example, for neurogenic or muscular GRNs [11,16]. Since most trichome enhancers are often active in the very same population of cells, with highly similar dynamics, it is surprising to observe such diversity in their motif compositions. There are four enhancers restricted to dorsal trichome cells, but again they accommodate different motif compositions, with *EminB *and *4702B*, which contain blue motifs, versus *cyrA *and *31559*, which do not. These data thus indicate that trichome enhancers display diverse distributions of functional motifs, supporting that distinct *cis*-regulatory architectures drive highly similar spatio-temporal expression.

### Flexibility in *cis*-regulatory motifs among enhancers versus across species

Although highly constrained sequences, such as the interferon-β enhanceosome, do not seem widely spread [20], developmental enhancers may yet require some 'grammar' for motif positioning [23] - for example, with an optimal pair-wise spacing of motifs [64] that could reflect the cooperative binding of TFs. For trichome enhancers we did not detect any obvious bias in the number or respective arrangement of the *cis*-regulatory motifs they rely on (Figure S2 in Additional file 1). Likewise, recent results from the analysis of *Drosophila *cardiac enhancers support that similar expression patterns can be generated from divergent compositions and positioning of motifs [10,65].

That several different inputs lead to similar enhancer outputs does not, however, formally rule out the existence of constraints, even though they are not detected by 'horizontal' comparison of different enhancers within the same species. An independent way to evaluate this possibility is to look at the evolution of individual regulatory regions throughout species [15,21]. Across Drosophilidae, trichome enhancers often display similar numbers and organization of *cis*-regulatory motifs (Figure 8; Figure S6 in Additional file 1). Furthermore, besides turnover of some motifs, svbF7, blue and yellow motifs are often embedded within short-sized islands of high evolutionary conservation, when compared to neighboring sequences (Figure 8). Similar strong evolutionary conservation was also noticed for the binding site of Twist [62] and its partner TFs [15], although these studies did not examine evolution of the detailed pattern of motif positioning. These data therefore suggest that despite diverse arrangements of motifs, patterns of evolutionary conservation likely represent the signature of functional constraints that locally shape the architecture of individual enhancers.

## Materials and methods

### Fly strains and transgenic constructs

We used *btd*, *svb^1 ^*or *svb^R9 ^*[30,31] and *pri^1 ^*[34] stocks kept over green fluorescent protein (GFP) balancers. To delineate the epidermal enhancer of *sn *and *sha*, transgenic lines were initially generated using P-element-mediated transformation (Fly Facility) and at least three independent insertions were analyzed for each construct. We then switched to the PhiC31 system (Bestgene, Chino Hills, CA, USA) to quantify effects of mutations, with all constructs integrated at the same location (*zh-86F*), except for *sha1*, *sha3 *and *snE1*, for which mutant versions were assayed in P-elements for homogeneity (Additional file 3). Genomic regions were amplified and cloned into pCasper or pAttB lacZ derivatives. QuikChange II XL site-directed mutagenesis (Agilent Technologies, Santa Clara, CA, USA) was used to introduce point mutations in enhancers, or CCGCCGGCGG stretches for linker scanning of *Emin*. All constructs were verified by sequencing. Details (genomic position) of the CRM are given in Additional file 3.

### Embryo staining

Dig- or biotin-labeled antisense RNA probes were used for *in situ *hybridization following standard protocols and embryos imaged using a Nikon Eclipse90i microscope. For immunodetection of lacZ reporter expression, 10- to 14-h embryos were stained using anti-β-galactosidase (1/1,000; Cappel, MP Biomedical, Solon, OH USA) and Alexafluor488 (Molecular Probes, Life Technologies, Carlsbad, CA, USA). Pictures were taken with a Leica SP2 confocal microscope, using the same settings to allow quantitative comparisons.

### Microarrays

We hand selected 13- to 15-h *svb^R9 ^*or *pri^1 ^*embryos using GFP balancers. We subjected 200 embryos to trizol (Invitrogen, Life Technologies, Carlsbad, CA, USA) extraction and RNA quality was monitored using Agilent Chip. Five independent samples of each genotype were used for microarrays (Affymetrix, Santa Clara, CA, USA; IGBMC, Strasbourg, France). Data extraction and normalization were performed using Affymetrix software and statistical analyses with R. A more than two-fold difference in expression levels between mutant genotypes was the most efficient criterion to retrieve Svb downstream genes (with a false discovery rate of 0.01 for *pri*). The top 150 genes down-regulated in both *pri *and *svb *mutants defined the set of Svb-regulated genes. One-hundred genes showing irrelevant variation of their expression (*P*-value > 0.8, false discovery rate >0.99) were used as a negative control set. The data discussed in this article have been deposited in NCBI's Gene Expression Omnibus [66] and are accessible through GEO series accession number GSE48997. Details are given in Additional file 3.

### ChIP-seq

A *svb *rescue construct (RSQ8) [34] was used for ChIP-seq experiments. It expresses a Svb-GFP protein under the control of two *svb *enhancers (medial and proximal) driving specific expression in epidermal trichome cells. Stocks were expanded to fill three population cages. Adults were allowed to lay eggs for 2 hours on apple juice plates covered with yeast. Embryos deposited on the plates were aged for 12 h at 25°C. Chromatin was collected from approximately 100 mg of whole embryos for each replicate chromatin collection. ChIP was done with an anti-GFP antibody as described [8]. Data presented are from two independent replicates. Peaks were called for single replicates using MACS *P *< 0.00001 for downstream computational analyses. MACS was used to call loose criteria peaks for two replicates of RSQ8 12- to 14-h embryos. Those peaks were then used for an IDR (Irreproducible Discovery Rate) analysis (IDR = 0.02). DNA sequencing libraries were generated with Nextera DNA Sequencing Library kits (VWR, Radnor, PA, USA) Details are given in Additional files 3 and 4. The data discussed in this publication have been deposited in NCBI's Gene Expression Omnibus [66] and are accessible through GEO series accession number GSE48791.

### Motif detection and genome analysis

Detection of motifs enriched in Svb-dependent and Svb-independent epidermal genes was performed using cisTargetX [40]. For *de novo *motif discovery, genomic sequences of enhancer and negative regions were processed through a C++ program and statistical operations performed within the R software, as described [47]. To compute the cross-correlation between conserved motif instances and Svb ChIP-Seq data, we defined a 10 kb window centered around each ChIP peak, collected distances of each motif to the peak center and plotted these values using a 500 bp bin. In the cases of Svb-regulated and control genes, each ChIP peak was associated with the nearest transcription start site. Further details are available in Additional files 3 and 4.

## List of abbreviations used

bp: base pair; BS: binding site; ChIP: chromatin immunoprecipitation; CRM: *cis*-regulatory module; GFP: green fluorescent protein; GRN: gene regulatory network; TF: transcription factor.

## Competing interests

The authors declare that they have no competing interests.

## Authors' contributions

SA, FP and SP designed the experiments, MS, HR and VH statistical analyses; DM, MS, IF, RS, JZ, IG, YL, PF, SA and SP performed the experiments and are listed according to their contributions. SP and FP wrote the paper and all authors commented on the manuscript. All authors read and approved the final manuscript.

## Supplementary Material

Additional file 1 **Supplementary figures**. Click here for file

Additional file 2 **Legends to the supplementary figures**. Click here for file

Additional file 3 **Supplementary information (details of experimental procedure, constructs, and so on)**. Click here for file

Additional file 4 **bed files (BS prediction, Chip-seq)**. Click here for file
