# Supplementary material for: Genome-wide analyses of Shavenbaby target genes reveals distinct features of enhancer organization
Source: Genome Biol. 2013 Aug 23;14(8):R86. doi: 10.1186/gb-2013-14-8-r86 (PMC4053989; doi:10.1186/gb-2013-14-8-r86)
Supplement: Additional file 1 — Supplementary figures. [file gb-2013-14-8-r86-S1.PDF]

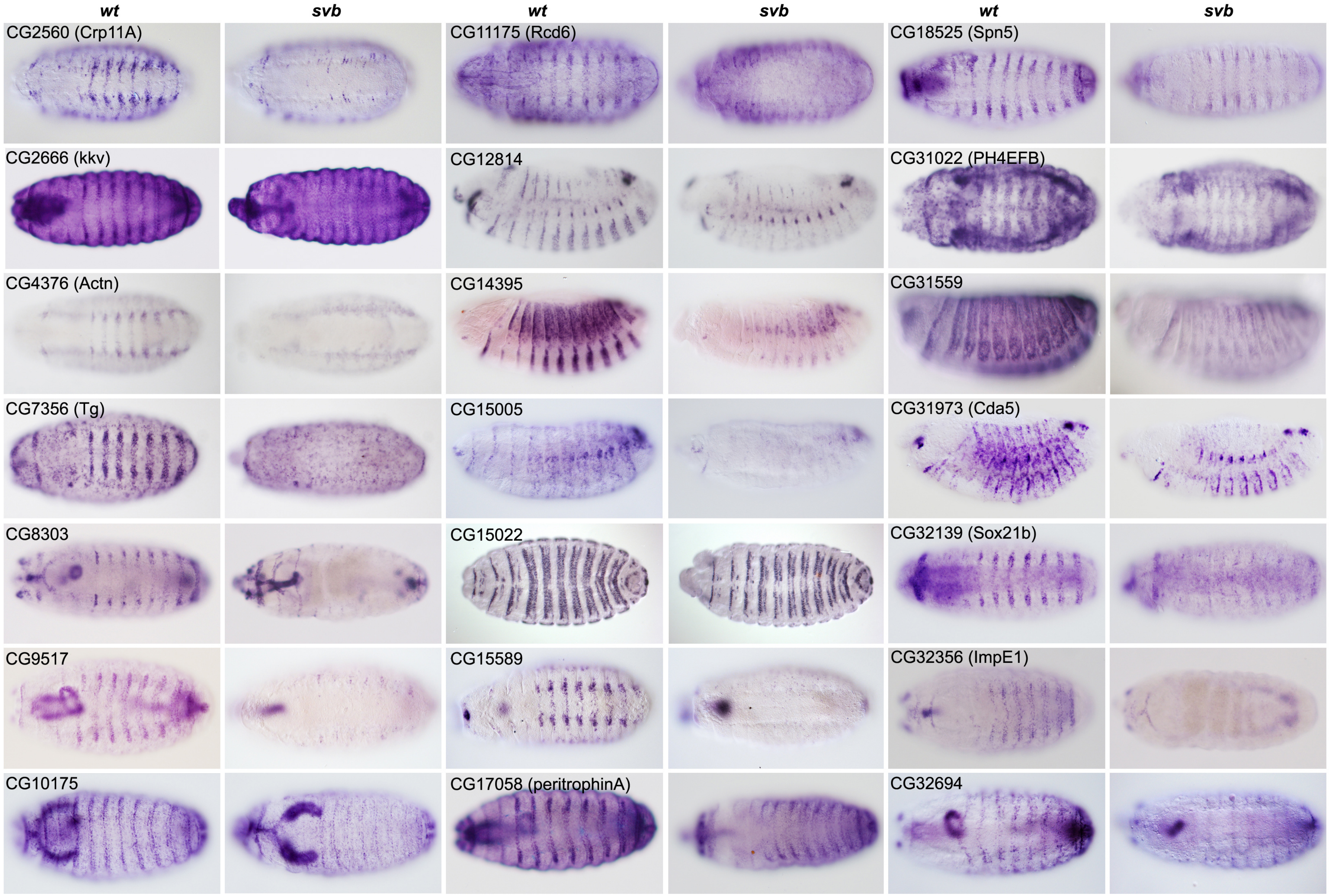

Menoret *et al.;* Figure S1A

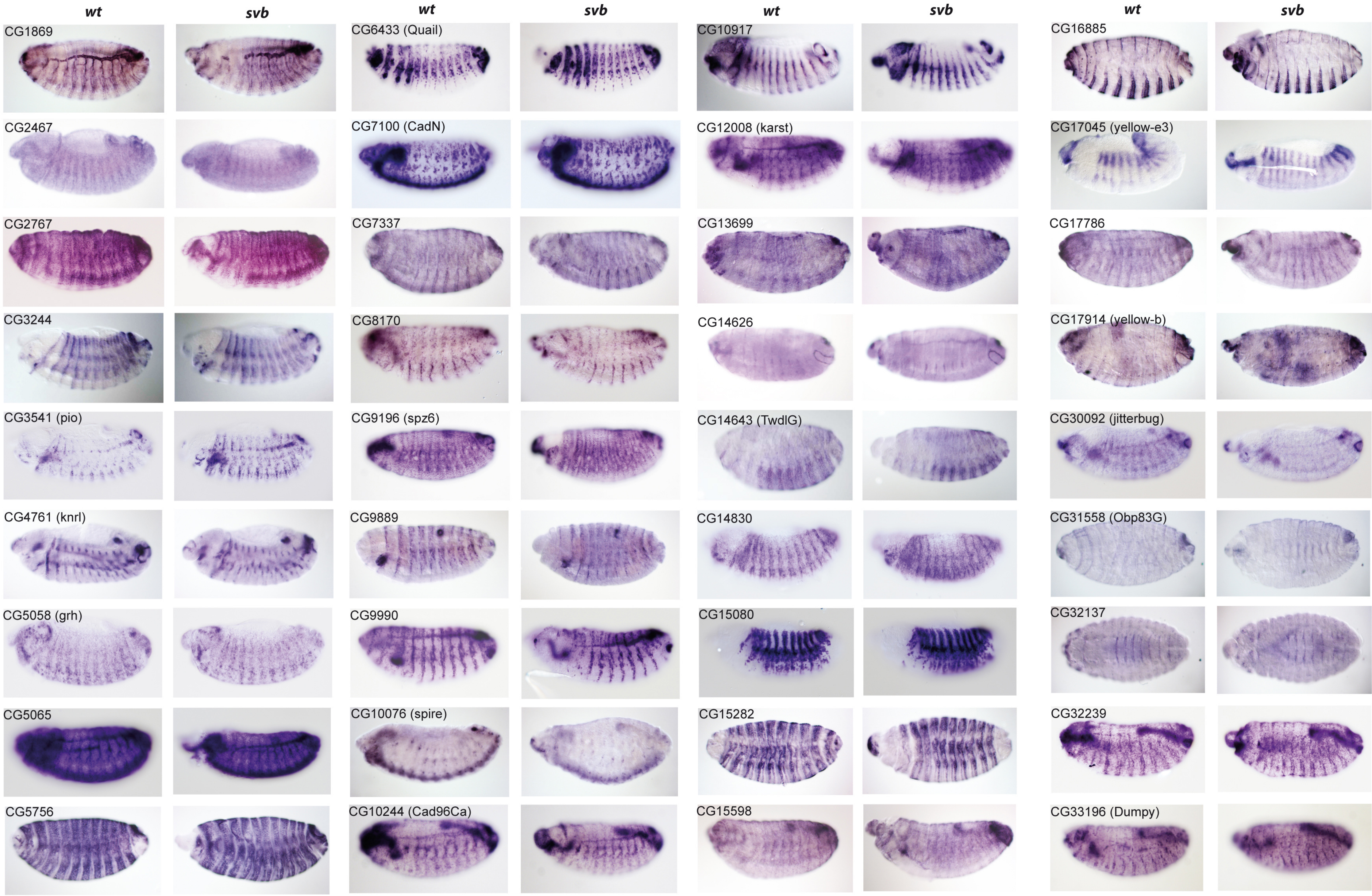

Menoret *et al.;* Figure S1B

cis-TargetX motifs detection

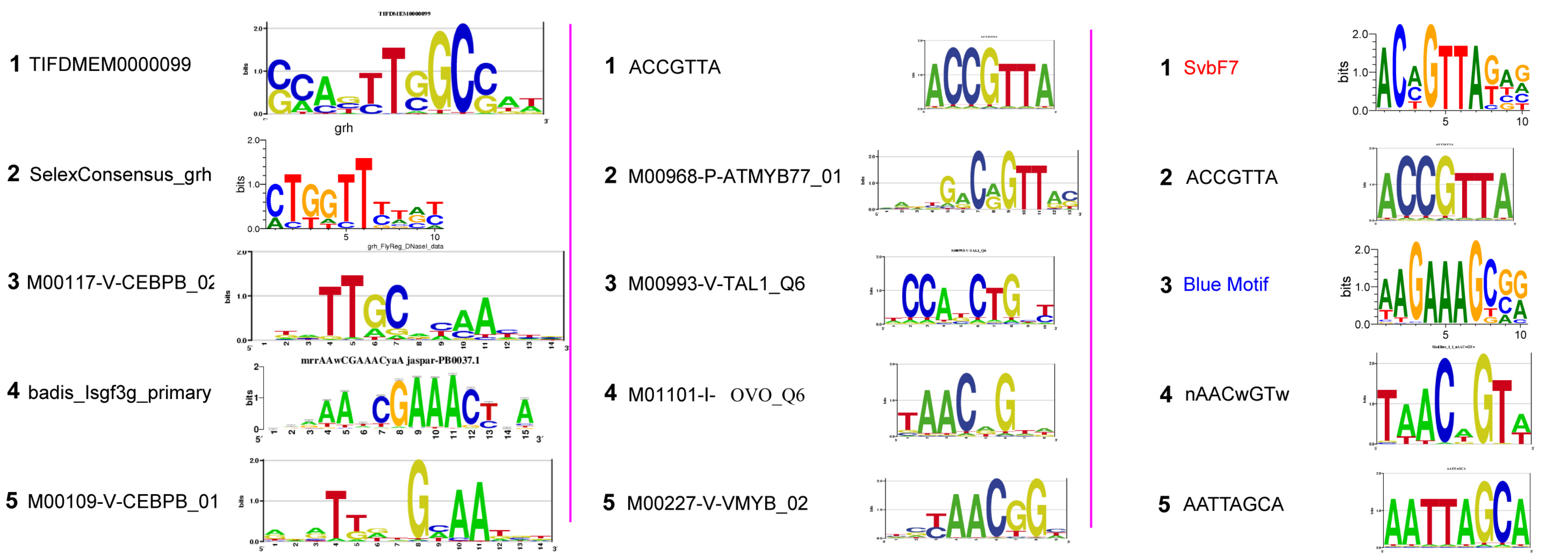

Ranking of genomic predicted regions

CisTargetX predicted CRMs	dyl1	15589	cyrA	snE5	dyl2	pmin	f2	32159	Emin	sox21b	sn-enh1	sha3	snH5	snB2	nyo3	snE1	sha-intron	EminB	f5	sha2	f4	cyrB	snP	nyo1	snE4
OvoQ6	1	3	4	5	8	ND	9	ND	ND	13	21	23	24	32	38	43	50	ND	55	63	73	77	79	80	82
svbF7	11	1	2	52	15	21	ND	24	26	ND	104	34	ND	58	7	38	ND	55	ND	10	ND	ND	ND	97	ND

Additional epidermal CRMs identified

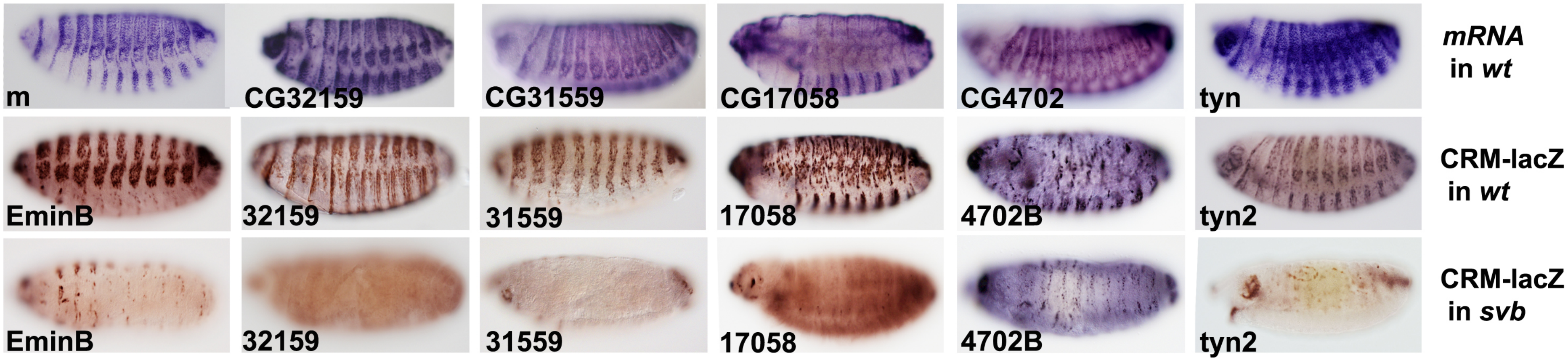

Menoret *et al.;* Figure S1C

rank	name	score
2	SvbF7	8.95
4	OvoQ6	8.09
5	ovo_SOLEXA	7.53
6	ovo_SANGER	7.49
9	ovo_FlyReg	5.56

PWM

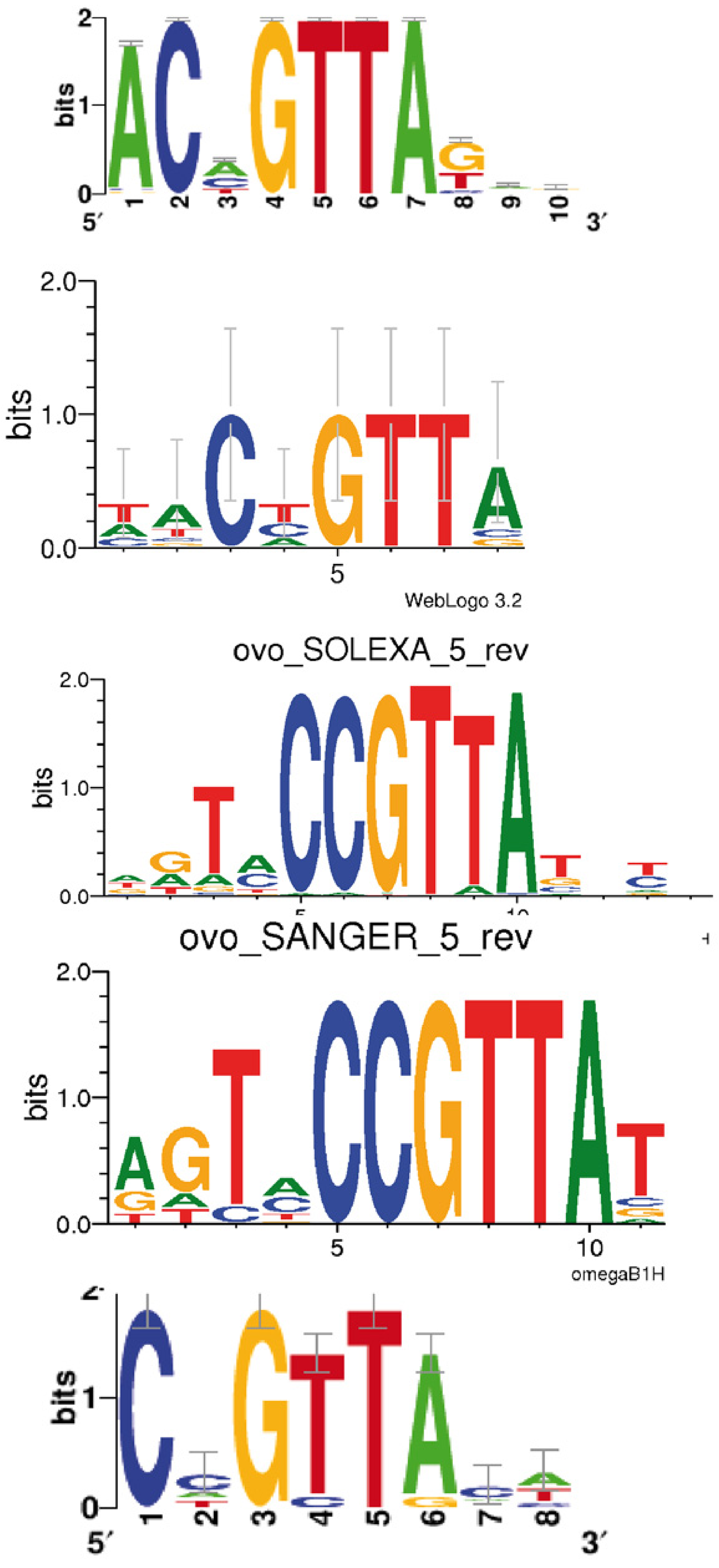

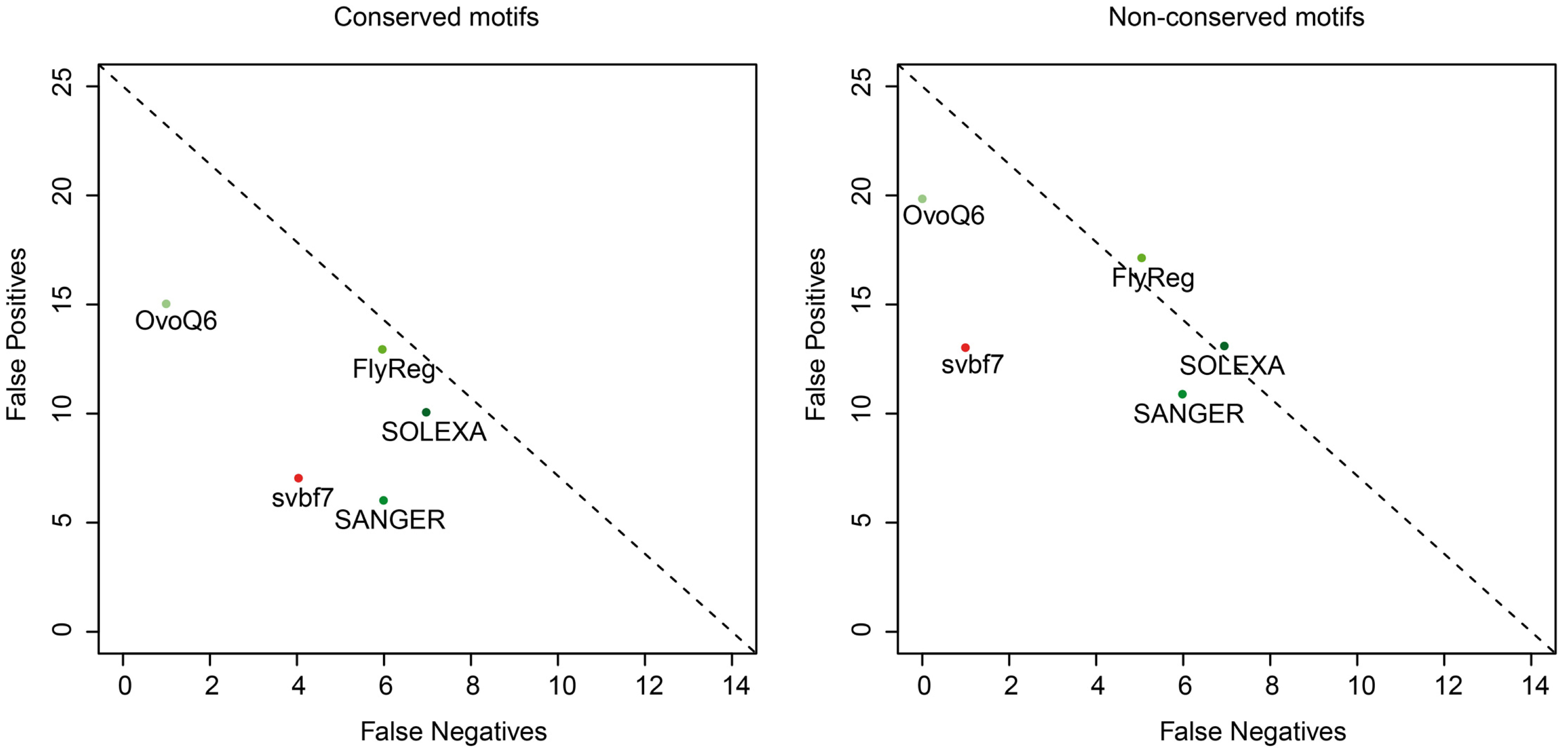

Menoret *et al.;* Figure S1D

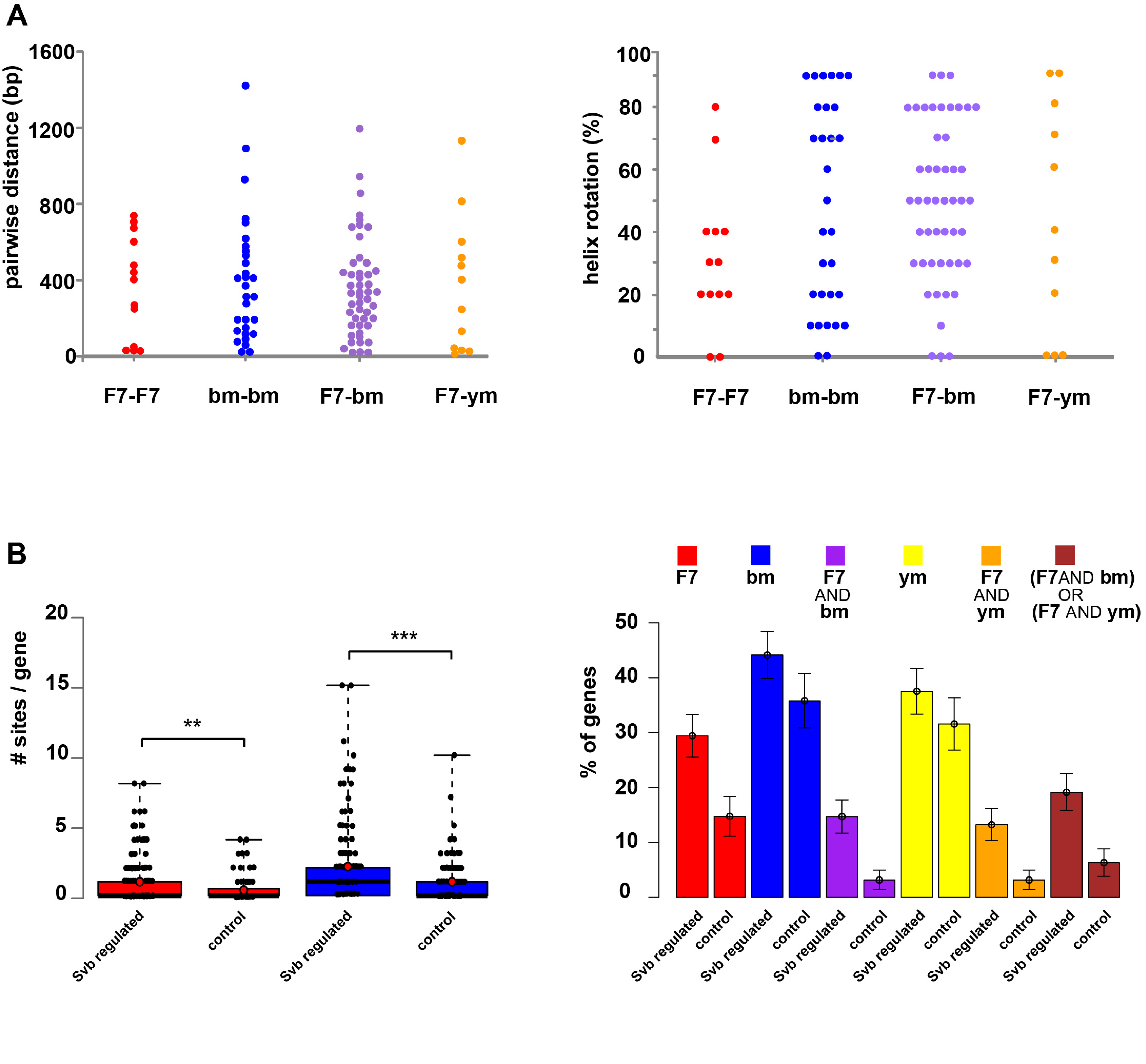

Menoret *et al.;* Figure S2

Gene symbol	Representative Public ID	Protein features/putative domains	epidermal expression	additional expression #	svb mutant microarrays (% of wt)	pri mutant microarrays (% of wt)	**validated in svb mutant (in situ)**	**validated in svb ectopic (in situ)**	**svbF7**	**blue motif**	**yellow motif**	ChIP peaks in 5kb (12-14h)	ChIP intensity (12-14h)	ChIP peaks in 5kb (8-10h)	**reference**
CG15370	CG15370	unknown	no	ubiquitous	13,1	2,1	no	ND	no	yes	no	yes	502	yes	this work
sha	CG13209	actin binding	stripes		24,6	6,6	yes	yes	yes	yes	yes	yes	92,92	yes	ref 1
CG14395	CG14395	PH like domain	stripes		41,2	3,0	yes	yes	yes	yes	yes	yes	135,174	yes	this work, FigS1
CG4386	CG4386	trypsin like protease	no	trachea	43,5	11,3	no	ND	no	yes	yes	no		no	this work
CG15818	CG15818	C-type lectin domain	no	midgut	47,9	17,8	no	ND	no	yes	no	no		yes	this work
m	CG9369	ECM	stripes		48,5	6,2	yes	yes	yes	yes	no	yes	173,768,69	yes	ref 1
CG32159	CG32159	Cuticle formation	stripes		50,1	12,6	yes	yes	yes	yes	yes	yes	56,789,69,54	yes	ref 2
nyo	CG1499	ECM	stripes	all gut	54,7	15,3	yes	yes	yes	yes	yes	yes	500	yes	ref 1
CG4914	CG4914	trypsin domain	stripes	hindgut	55,9	21,7	yes	ND	yes	yes	yes	yes	418,418	yes	this work, FigS4
neyo	CG7802	ECM	stripes	all gut	56,1	17,3	yes	yes	no	yes	no	yes	59,14	yes	ref 1
mey	CG12063	ECM	stripes		57,1	7,8	yes	yes	yes	yes	yes	yes	654	yes	ref 1
CG16798	CG16798	unknown	stripes		57,1	18,4	yes	yes	no	yes	yes	yes	140	yes	ref 1
CHOp24	CG3564	protein secretion	stripes	salivary gland	59,0	27,0	ND	ND	no	yes	no	no		yes	
CG11905	CG11905	unknown	stripes	trachea	59,3	20,1	yes	no	yes	yes	no	no		yes	this work, FigS4
CG14356	CG14356	unknown	no	foregut	60,3	27,1	ND	ND	yes	no	yes	no		no	
CG17211	CG17211	von Willebrand factor type C domain	ND	ND	60,5	26,4	ND	ND	no	yes	yes	yes	162	yes	
CG17780	CG17781	unknown	no	hindgut & anal pad	61,0	27,1	no	ND	no	yes	yes	no		no	this work
mwh	CG13913	actin binding	stripes		61,1	25,6	yes	yes	no	no	no	yes	333	yes	ref 1
CG30283	CG30283	trypsin like protease	stripes	atrium & adult eve PR	62,0	18,3	ND	ND	no	yes	no	no		yes	
CG4500	CG4500	bubblegum like domain	ND	ND	65,2	30,3	ND	ND	no	no	yes	yes	956	yes	
spz6	CG9196	Toll signalling pathway	stripes		66,9	16,5	no	no	no	yes	no	no		yes	this work, FigS1
CG1273	CG1273	unknown	stripes	all gut & trachea	67,2	20,8	yes	yes	yes	yes	yes	yes	98,98,52,84,	yes	this work, FigS4
CG6785	CG6785	unknown	ND	ND	67,7	21,4	ND	ND	no	no	no	yes	77	yes	
CG10591	CG10591	unknown	stripes		67,8	21,7	yes	yes	yes	no	yes	yes	86	yes	this work, FigS4
CG13698	CG13698	unknown	stripes	gut & salivary gland	68,4	29,3	yes	yes	yes	yes	yes	yes	51,219,1214	yes	this work, FigS4
CG9514	CG9514	GMC oxidoreductase	stripes		69,0	22,4	yes	yes	yes	yes	yes	yes	175,111	no	this work, FigS4
CG17562	CG17562	fatty acyl CoA reductase	no	oenocytes	69,2	25,3	no	ND	no	no	no	no		no	this work
f	CG5424	actin binding	stripes		70,3	26,0	yes	yes	yes	yes	yes	yes	106,122,59,136,57	yes	ref 1
CG9184	CG9184	unknown	stripes	corpus cardiacum	70,9	15,7	yes	yes	yes	yes	yes	yes	100,59,303,	yes	this work, FigS4
CG12075	CG12075	PH like domain	stripes	gut & salivary gland	71,5	33,4	yes	yes	yes	yes	yes	yes	139,253,135,54,50,186	yes	this work, FigS4
tyn	CG17131	ECM	stripes		72,9	22,2	yes	yes	yes	yes	yes	yes	291,147,200,66,67,99,252	no	ref 1
CG4678	CG4678	metallocarboxypeptidase	no	foregut & anal pad	73,4	36,3	ND	ND	yes	no	yes	yes	52	yes	
CG13616	CG13616	unknown	no	intestine & anal pad	73,4	12,7	ND	ND	no	yes	yes	no		no	
CG14756	CG14756	unknown	no	salivary gland	74,0	12,3	ND	ND	no	yes	no	no		no	
CG4666	CG4666	hot-dog domain	stripes	post spiracle	76,5	24,5	yes	yes	no	yes	yes	yes	66,136,	yes	this work, FigS4
CG4686	CG4686	unknown	ND	ND	77,0	33,7	ND	ND	no	no	no	no		no	
CG13082	CG13082	ketohexokinase	stripes	all gut	77,7	20,7	ND	ND	yes	yes	no	no		yes	
CG7173	CG7173	serine protease inhibitor	stripes		77,8	11,6	yes	yes	no	no	yes	yes	73,65,	yes	this work, FigS4
CG1632	CG1632	trypsin like protease	ND	ND	78,0	18,9	ND	ND	no	no	yes	yes	74,477,86,104,92	yes	
Orct	CG6331	Major Facilitator Superfamily	no	midgut & fat body	78,1	35,5	ND	ND	no	no	no	yes	70	no	
mas	CG15002	chymotrypsin, endopeptidase	stripes	foregut & hindgut & trachea	78,1	31,7	yes	yes	no	yes	no	yes	57	yes	this work, FigS4
CG5039	CG5039	unknown	ND	ND	78,6	12,6	ND	ND	no	yes	yes	yes	109	yes	
CG12017	CG12017	unknown	stripes		78,6	18,0	yes	yes	yes	yes	yes	yes	56,223,72,84,	yes	this work, FigS4
CG5873	CG5873	redox process	stripes	all gut & trachea	78,7	36,1	ND	ND	yes	yes	no	yes	281	yes	
CG8420	CG8420	unknown	stripes		79,3	15,0	yes	no	yes	yes	yes	yes	190,86,170,	yes	this work, FigS4
CG11200	CG11200	metabolic process	stripes	trachea	79,8	32,0	ND	ND	no	no	no	yes	104	yes	
Cyp301a1	CG8587	redox process	stripes	trachea	80,3	34,3	ND	ND	yes	no	no	no		no	
CG42331	CG6879	redox process	ND	ND	80,4	9,8	ND	ND	yes	yes	yes	yes	97,64,64,99	yes	
CG13822	CG13822	GILT domain, thiol reductase	stripes	lymph gland	80,8	34,3	yes	yes	yes	no	yes	yes	181	yes	this work, FigS4
CG12009	CG12009	chitin metabolic process	no	trachea	82,4	17,3	ND	ND	yes	yes	no	yes	53,503	yes	
CG8239	CG8239	isoprenoid biosynthesis	stripes	hindgut & anal pad	82,9	12,7	no	ND	no	no	no	no		no	this work
snRNP-U1	CG5454	mRNA splicing	no	ubiquitous	83,2	38,9	ND	ND	no	yes	no	no		yes	
CG1140	CG1140	ketone body catabolism	no	midgut & fat body	83,4	39,7	ND	ND	no	no	yes	yes	74	no	
dyl	CG15013	ECM	stripes	foregut & hindgut	85,3	16,1	yes	yes	yes	yes	yes	yes	57,58,174,85,498,528,285,57	yes	ref 1
Hr46	CG33183	ecdysone pathway	no	ubiquitous	85,5	39,9	ND	ND	yes	yes	yes	yes	47,54,262,108,460,60,190,70	yes	
vri	CG14029	tracheal system development	stripes	hindgut	85,6	30,5	no	ND	yes	yes	yes	yes	78,187,54	yes	
wus	CG9089	ECM	stripes	all gut & trachea	86,1	12,6	no	ND	no	no	yes	yes	188	yes	this work

ImpE1	CG32356	ecdysone pathway	stripes	all gut	86,3	14,8	yes	yes	yes	yes	yes	yes	67,251	yes	this work, FigS1
Plip	CG10371	protein dephosphorylation	ubiquitous	midgut & hindgut	86,7	32,5	ND	ND	no	no	yes	yes	239	yes	
CG17672	CG11271	translation	no	ubiquitous	86,7	22,3	ND	ND	no	no	no	yes	573,102	yes	
CG32354	CG32354	unknown	stripes	all gut	86,8	42,3	ND	ND	no	yes	yes	no		yes	
CG8306	CG8306	redox process	stripes	foregut & hindgut	86,8	41,3	ND	ND	no	no	no	no		yes	
CG10585	CG10585	unknown	stripes	all gut	87,1	27,3	ND	ND	no	yes	yes	no		yes	
fw	CG1500	cell adhesion	no		87,2	30,5	ND	ND	no	yes	yes	yes	463,156	yes	
Obp99c	CG7584	sensory perception	no	fat body & amnioserosa	87,3	27,4	ND	ND	no	yes	yes	no		yes	
CG5525	CG5525	microtubule organization	no	muscle system & hindgut	87,4	31,5	ND	ND	no	yes	yes	no		no	
CG34007	Dm.2L.4959.0	unknown	ND	ND	87,5	25,6	ND	ND	no	no	no	yes	164	yes	
CG14470	CG14470	unknown	no	hindgut	87,5	39,1	ND	ND	no	no	no	no		no	
CG18249	CG18249	unknown	no	midgut & amnioserosa	87,6	28,4	ND	ND	no	no	no	yes	59	yes	
CG8386	CG8386	lateral inhibition	ubiquitous	salivary gland	87,9	41,5	ND	ND	no	no	no	no		no	
pwn	CG11101	EGF-like calcium binding	ND	ND	88,0	29,8	ND	ND	no	yes	yes	yes	51,51	yes	
PH4alphaSG1	CG31014	redox process	no	salivary gland	88,1	39,7	ND	ND	no	no	yes	no		no	
CG6415	CG6415	glycin catabolic process	no	fat body	88,2	37,1	ND	ND	no	yes	yes	yes	103	yes	
cher	CG3937	actin binding	stripes	muscle system	88,3	40,5	yes	no	yes	yes	yes	no		yes	this work, FigS4
CG2016	CG2016	unknown	stripes	all gut & trachea	88,4	22,9	ND	ND	no	yes	yes	no		no	
Tb	CG5480	Cuticle formation	stripes		88,6	21,5	yes	yes	no	yes	yes	yes	309,53	no	this work, FigS4
CG10932	CG10932	mitotic spindle organization	no	midgut	88,8	34,6	ND	ND	no	no	yes	no		yes	
scu	CG7113	ecdysone pathway	no	midgut	89,1	39,8	ND	ND	no	no	yes	no		no	
CG7860	CG7860	autophagic cell death	no	midgut & crystal cells	89,7	15,8	ND	ND	no	yes	no	no		yes	
CG9356	CG9356	unknown	no		89,7	39,8	ND	ND	no	no	no	no		yes	
Hmgs	CG4311	hydroxymethylglutaryl-CoA synthase	stripes	foregut & hindgut	90,0	28,7	yes	no	no	no	yes	yes	286	yes	this work, FigS4
CG11836	CG11836	proteolysis	ubiquitous		90,3	32,3	ND	ND	no	no	no	yes	111	no	
CG9503	CG9503	redox process	no	dorsal trunk	90,4	22,4	ND	ND	no	no	yes	yes	256	yes	
CG1837	CG1837	apoptotic cell clearance	no	ubiquitous	90,4	39,5	ND	ND	yes	no	no	yes	80,66	yes	
tw	CG12311	somatic muscle development	ND	ND	90,5	38,0	ND	ND	no	no	no	no		yes	
PH4alphaEFB	CG31022	procollagen dioxygenase	stripes	muscle system & plasmatocytes	90,6	40,5	yes	yes	yes	yes	yes	yes	540,78	yes	this work, FigS1
amd	CG10501	chitin metabolic process	ND	ND	90,6	38,1	ND	ND	yes	no	yes	yes	261,60,	yes	
Rab23	CG2108	GTPase, planar polarity	stripes	foregut	91,5	31,9	yes	yes	no	yes	no	yes	289	yes	this work, FigS4
Smn	CG16725	neuromuscular junction	no	gonad	91,5	37,6	ND	ND	no	no	no	no		yes	
Pros28.1	CG3422	protease	no	ubiquitous	91,6	28,1	ND	ND	yes	no	no	yes	204,185	yes	
CG9175	CG9175	unknown	no	midgut & hindgut& salivary gland	91,6	40,9	ND	ND	no	no	no	no		yes	
Rlc1	CG9378	translation	ND	ND	91,8	45,2	ND	ND	no	yes	no	no		yes	
Lip4	CG6113	lipid metabolism	no	amnioserosa	91,9	44,5	ND	ND	no	no	yes	yes	240	no	
CG7840	CG7840	lipid metabolism	no		91,9	35,2	ND	ND	yes	no	yes	no		no	
Gtp-bp	CG2522	protein secretion	stripes	midgut & hindgut& salivary gland	92,1	39,0	ND	ND	no	no	no	no		yes	
CG32250	CG32250	transport	ND	ND	92,2	41,7	ND	ND	no	no	no	no		no	
CG15506	CG15506	unknown	ND	ND	92,2	13,6	ND	ND	yes	yes	yes	yes	53,69	yes	
TRAM	CG11642	protein targeting to membrane	stripes	salivary gland	92,3	36,0	ND	ND	yes	no	yes	no		yes	
CG6704	CG6704	unknown	no	yolk nuclei	92,5	16,3	ND	ND	no	yes	yes	no		no	
CG17218	CG17218	tracheal system development	stripes	all gut & anal pad	92,5	40,8	ND	ND	no	no	yes	no		no	
CG4065	CG4065	unknown	no	midgut & muscle system	92,6	46,3	ND	ND	no	no	no	yes	322,66,	yes	
mRpL46	CG13922	unknown	no	midgut & muscle system	92,7	42,8	ND	ND	no	no	no	no		no	
CG6180	CG6180	unknown	no	midgut	92,7	44,0	ND	ND	no	yes	no	no		yes	
T-cp1	CG5374	protein folding	no	ubiquitous	92,9	41,1	ND	ND	yes	no	no	yes	72	no	
CG13585	CG13585	unknown	no	garland cell	93,0	45,3	ND	ND	no	no	yes	no		no	
nimB3	Dm.2L.8912.0	unknown	ND	ND	93,6	29,5	ND	ND	no	no	no	no		no	
CG2663	CG2663	transport	head	post spiracle	93,7	41,8	ND	ND	no	yes	yes	yes	208	yes	
CG11786	CG11786	unknown	no	dorsal trunk	93,8	27,0	ND	ND	no	no	no	yes	281	no	
rt	CG6097	synaptic activity	ND	ND	94,0	25,7	ND	ND	no	yes	no	yes	61	yes	
CG13627	CG13627	unknown	no	trachea	94,2	16,2	ND	ND	no	yes	yes	no		no	
Gmap	CG33206	protein targeting to Golgi	ND	ND	94,3	41,8	ND	ND	no	no	yes	no		yes	
CG6672	CG6672	transmembrane transport	ND	ND	94,4	42,0	ND	ND	no	yes	yes	no		yes	
CG4702	CG4702	unknown	stripes	all gut	94,5	16,9	yes	yes	no	yes	yes	yes	50,344	yes	ref 1
CG3831	CG3831	unknown	no	corpus allatum	94,5	37,3	ND	ND	yes	no	no	no		yes	

sec23	CG1250	secretory pathway	stripes	salivary gland	94,5	41,8	yes	ND	no	no	no	yes	136	yes	this work, FigS4
CG31559	CG31559	thioredoxin	stripes	foregut	94,8	14,3	yes	yes	no	yes	yes	yes	90,236,92	yes	this work, FigS1
CG11771	CG11771	proteolysis	no	all gut & muscle system	95,0	45,4	ND	ND	yes	no	yes	no		yes	
CG9095	CG9095	cell adhesion	stripes		95,4	36,9	yes	yes	yes	yes	yes	yes	89,265,200,111	yes	this work, FigS4
---	GM01028	unknown	ND	ND	95,6	42,4	ND	ND	no	no	no	yes	79	no	
CG32039	CG32039	unknown	no		95,7	26,7	ND	ND	no	no	yes	yes	122	yes	
CG1753	CG1753	cystein biosynthesis	no	midgut	96,0	26,3	ND	ND	no	yes	no	yes	154,93	yes	
CG4822	CG4822	unknown	ND	ND	96,3	43,6	ND	ND	yes	yes	no	yes	63	no	
CG8112	CG8112	unknown	no	yolk nuclei	96,8	30,2	ND	ND	no	yes	yes	yes	280	yes	
ect	CG6611	tube development	stripes	oesophagus & anal pad	96,9	19,7	yes	no	yes	yes	yes	yes	361	yes	this work, FigS4
CG15239	CG15239	unknown	stripes	salivary gland	97,0	19,4	ND	ND	yes	yes	yes	yes	166	yes	
CG9689	CG9689	unknown	stripes	oesophagus & post spiracle	97,1	43,7	ND	ND	no	yes	no	no		yes	
mRpL45	CG6949	translation & transport	no	ubiquitous	97,2	22,6	ND	ND	no	no	no	no		yes	
CG8213	CG8213	proteolysis	ND	ND	97,3	13,7	ND	ND	yes	yes	yes	no		yes	
CG2263	CG2263	phenylalanyl-tRNA aminoacylation	no	ubiquitous	97,4	46,9	ND	ND	no	yes	no	yes	109	yes	
Rpb8	CG11246	transcription	no	midgut	97,8	45,1	ND	ND	no	no	yes	yes	81	yes	
gua	CG6433	actin binding	stripes	all gut & lymph gland	97,8	43,1	no	no	yes	yes	yes	no		yes	this work, FigS1
CG11227	CG13630	proteolysis	ND	ND	97,8	47,0	ND	ND	yes	no	yes	no		no	
CG9205	CG9205	unknown	ND	ND	98,0	44,9	ND	ND	no	yes	yes	no		yes	
Nf-YA	CG3891	transcription & phagocytosis	no	ubiquitous	98,3	39,6	ND	ND	no	yes	no	yes	91	yes	
kar	CG12286	transmembrane transport	no	midgut & amnioserosa	98,3	42,8	ND	ND	no	no	yes	no		yes	
CG31717	CG31717	unknown	ND	ND	98,4	45,2	ND	ND	yes	yes	yes	no		no	
Past1	CG6148	endocytosis	no	midgut & salivary gland	98,4	46,6	ND	ND	no	yes	yes	yes	66	yes	
bw	CG17632	eye pigment biosynthesis	no	malpighian tubule & fat body	98,4	37,4	ND	ND	no	yes	no	no		yes	
mRpL51	CG13098	translation	no	midgut & muscle system	98,5	39,3	ND	ND	no	no	no	no		yes	
pk	CG11084	planar polarity	no	ubiquitous	98,6	45,5	ND	ND	yes	yes	yes	yes	190,178,125	yes	
CG5171	CG5171	trehalose biosynthesis	no	amnioserosa & yolk nuclei	98,9	37,8	ND	ND	no	yes	no	yes	351,159,153	yes	
CG13365	CG13365	unknown	no		98,9	43,2	ND	ND	no	yes	no	yes	518	yes	
CG5742	CG5742	neurogenesis	no	ubiquitous	98,9	43,8	ND	ND	no	no	yes	no		yes	
PKD	CG7125	intracellular signal transduction	ND	ND	99,0	46,5	ND	ND	no	yes	yes	yes	941,94	yes	
CG11127	CG11127	unknown	no		99,1	47,2	ND	ND	no	yes	yes	no		yes	
Fib	CG9888	centrosome organization	no	ubiquitous	99,1	49,2	ND	ND	no	no	no	no		yes	
CG30423	CG30423	unknown	ND	ND	99,1	43,0	ND	ND	yes	yes	yes	no		no	
CG3842	CG3842	redox process	stripes	foregut & anal pad	99,2	14,1	ND	ND	no	no	no	yes	213,160,74	yes	
CG15743	CG15743	phosphatidylinositol phosphorylation	no	salivary gland	99,9	44,9	ND	ND	no	no	yes	no		yes	

Menoret *et al.;* Figure S3

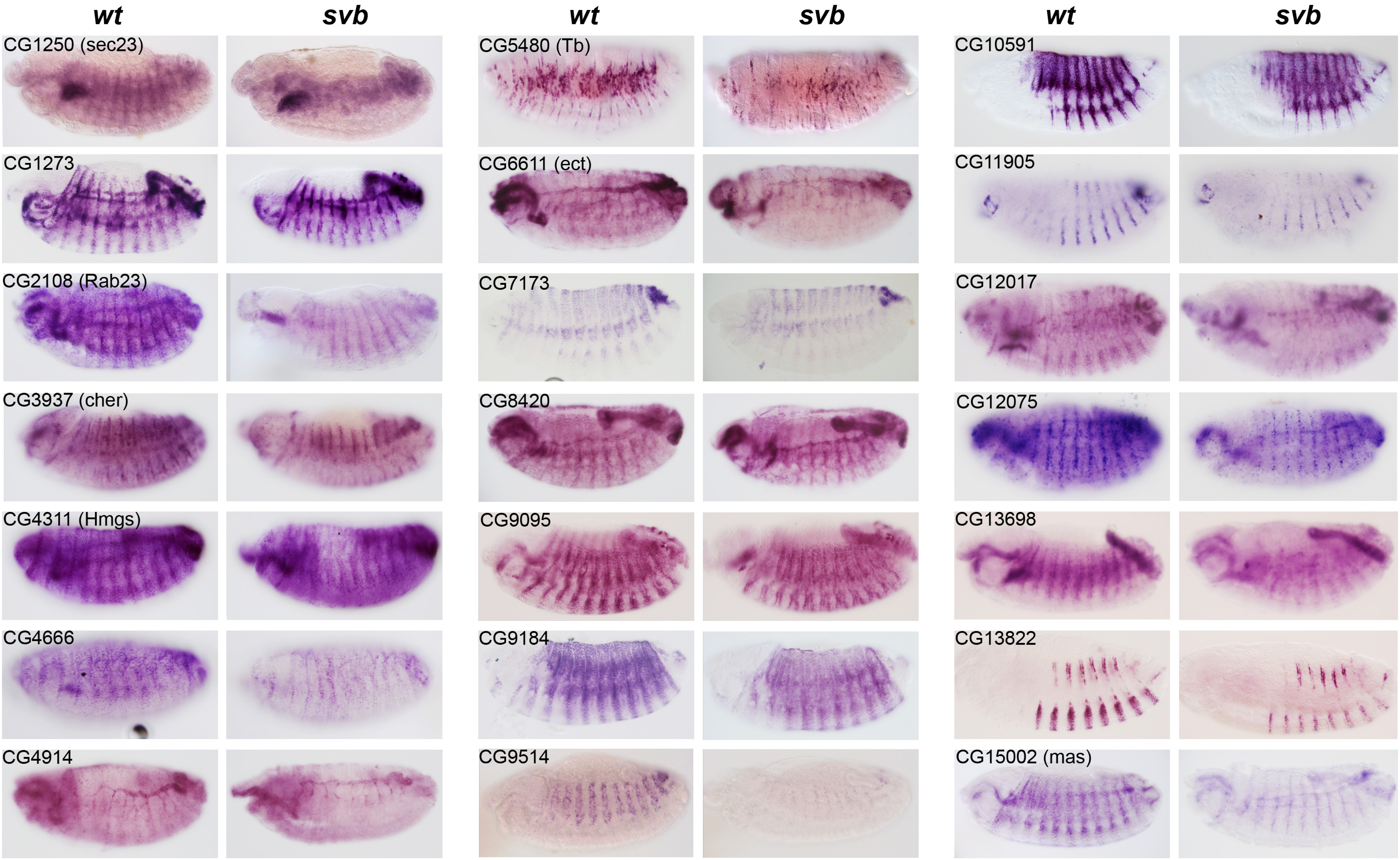

Menoret *et al.;* Figure S4

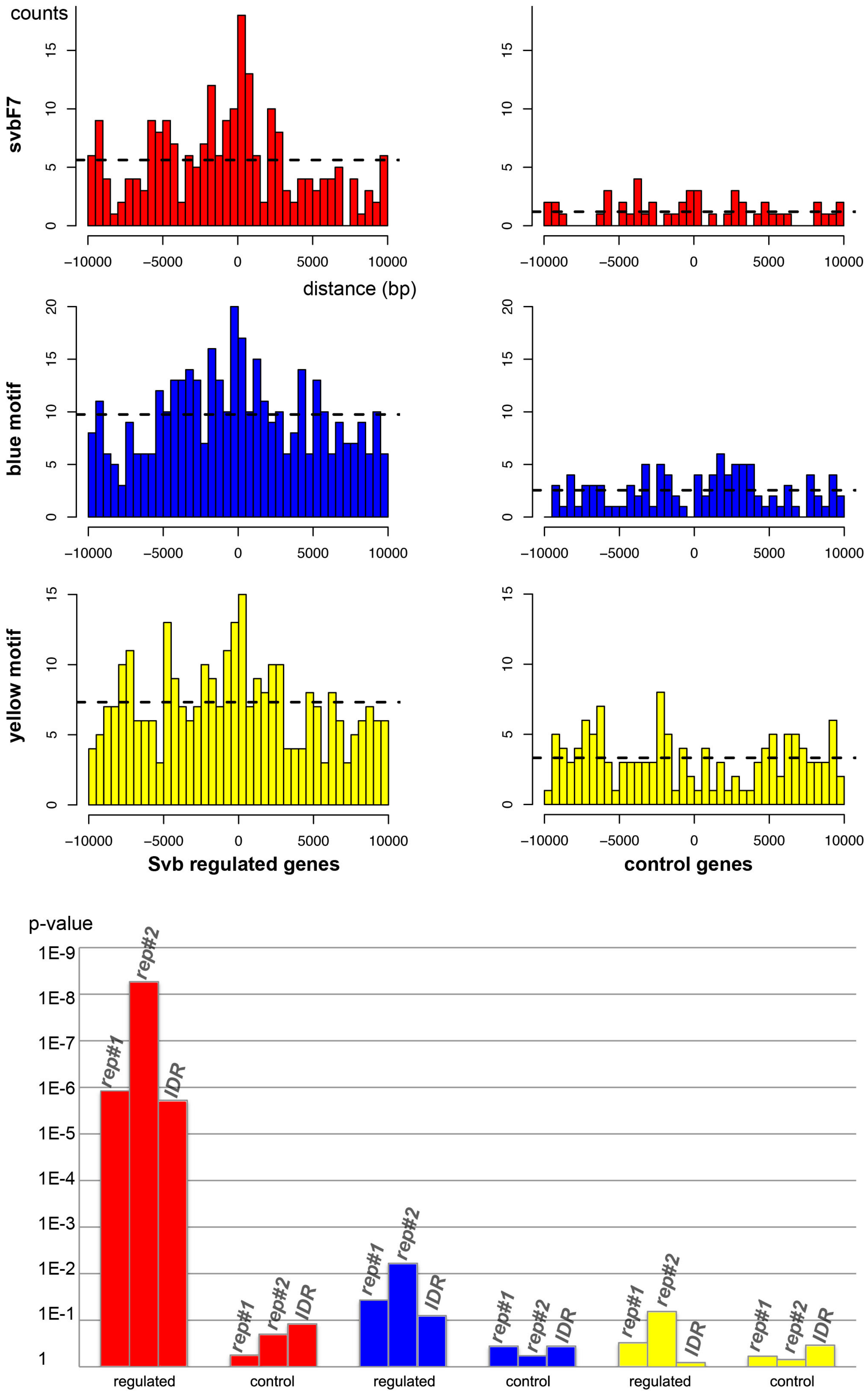

Menoret *et al.*; Figure S5

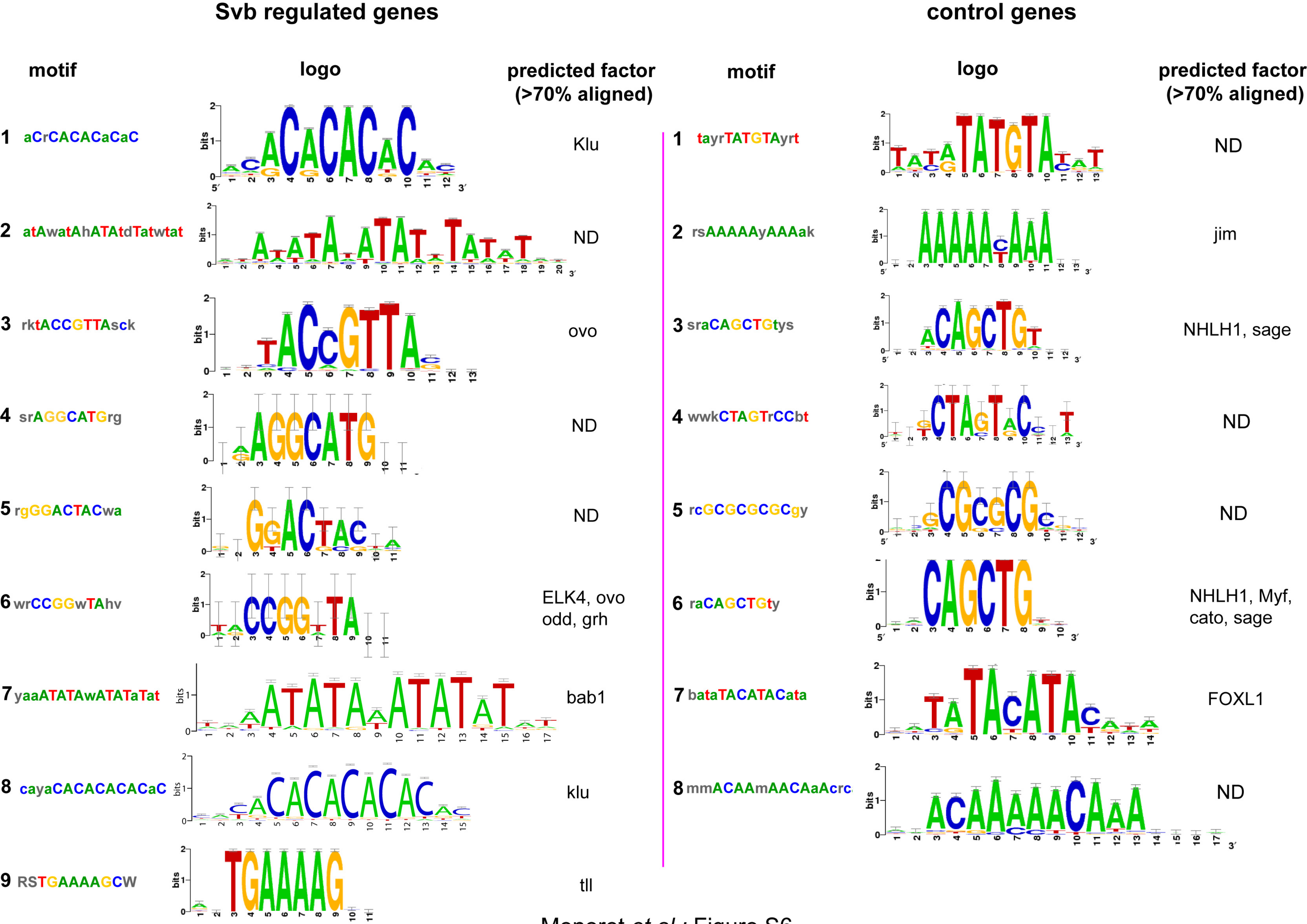

Menoret *et al.;* Figure S6

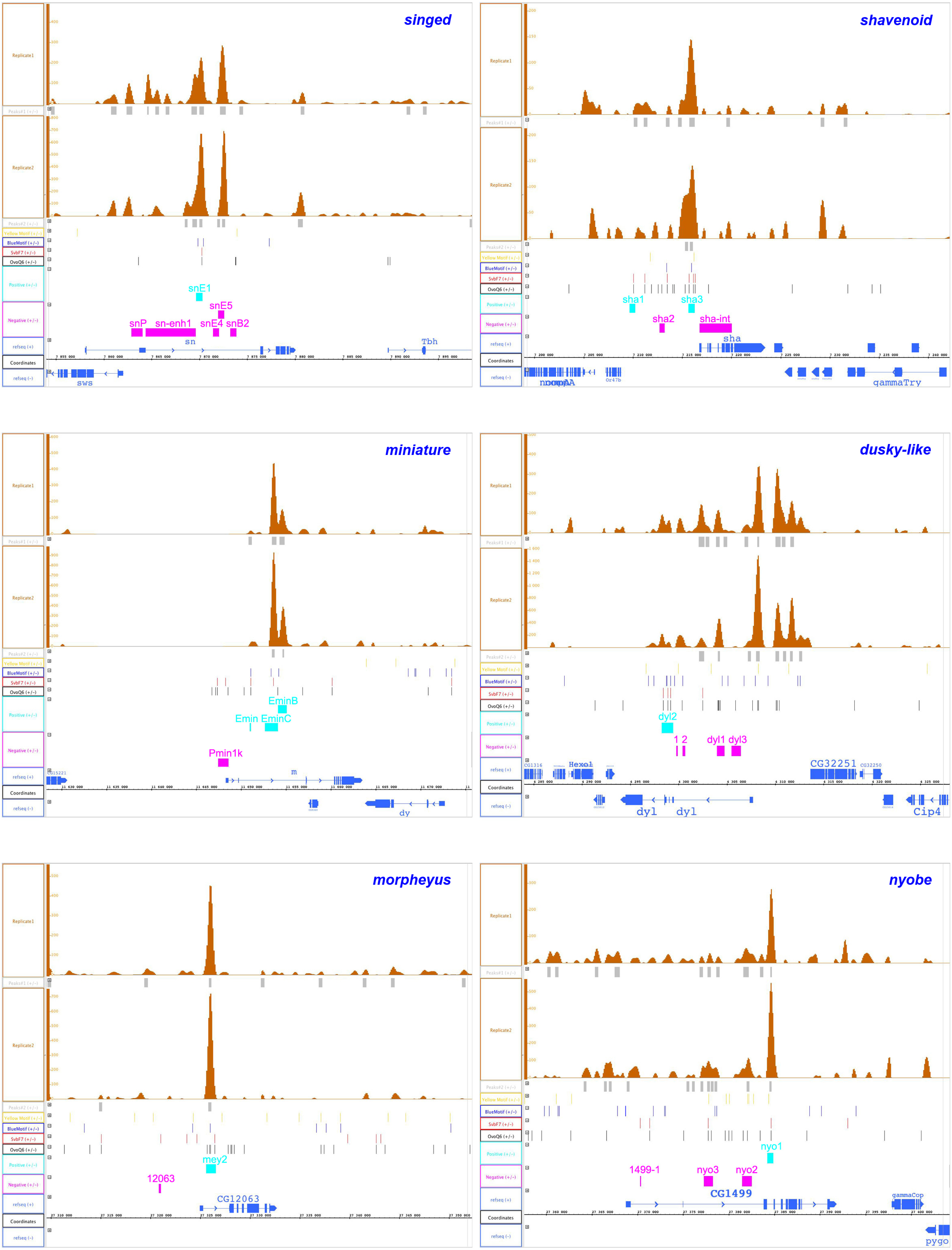

Menoret et al.; Fig S7A

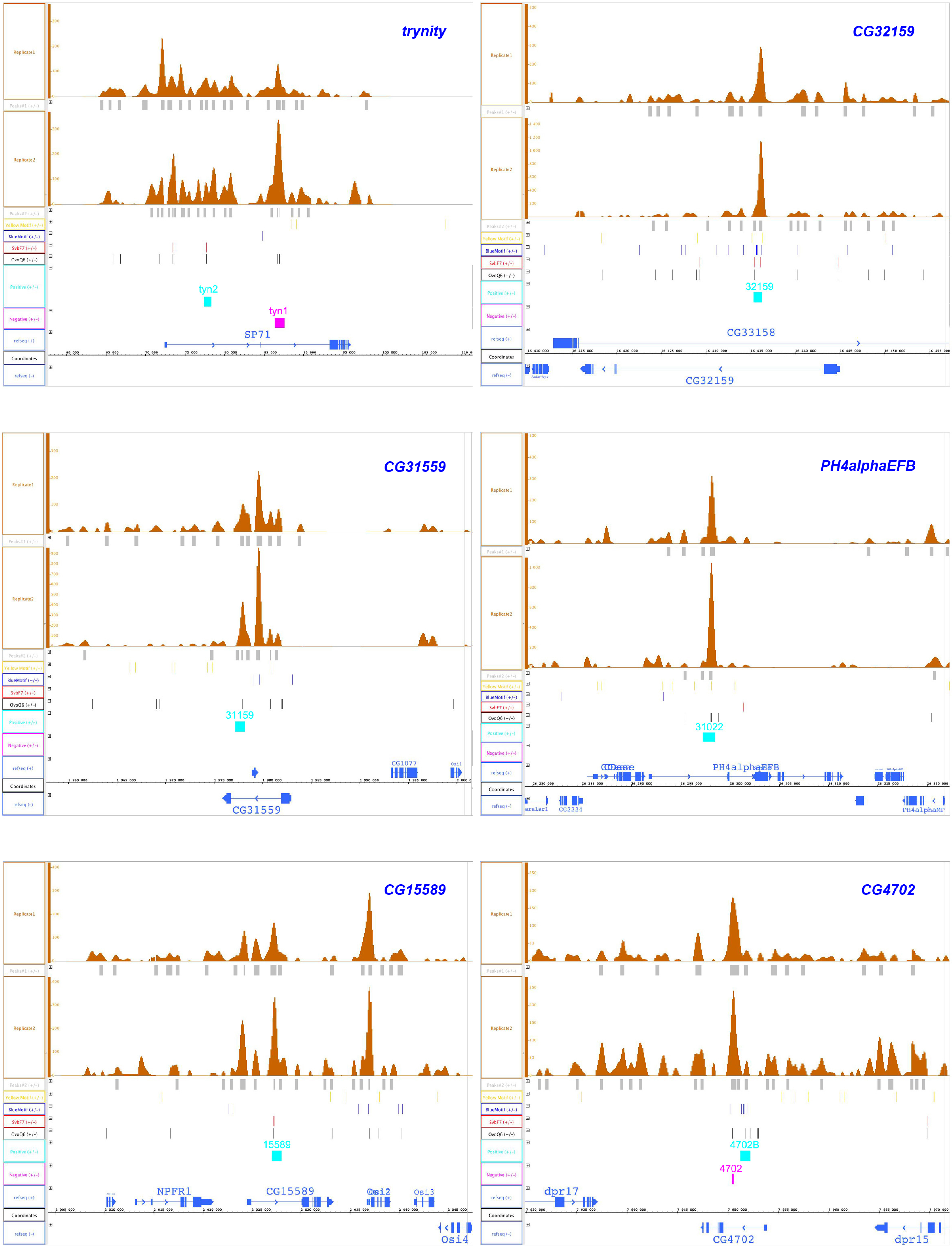

Menoret et al.; Fig S7B

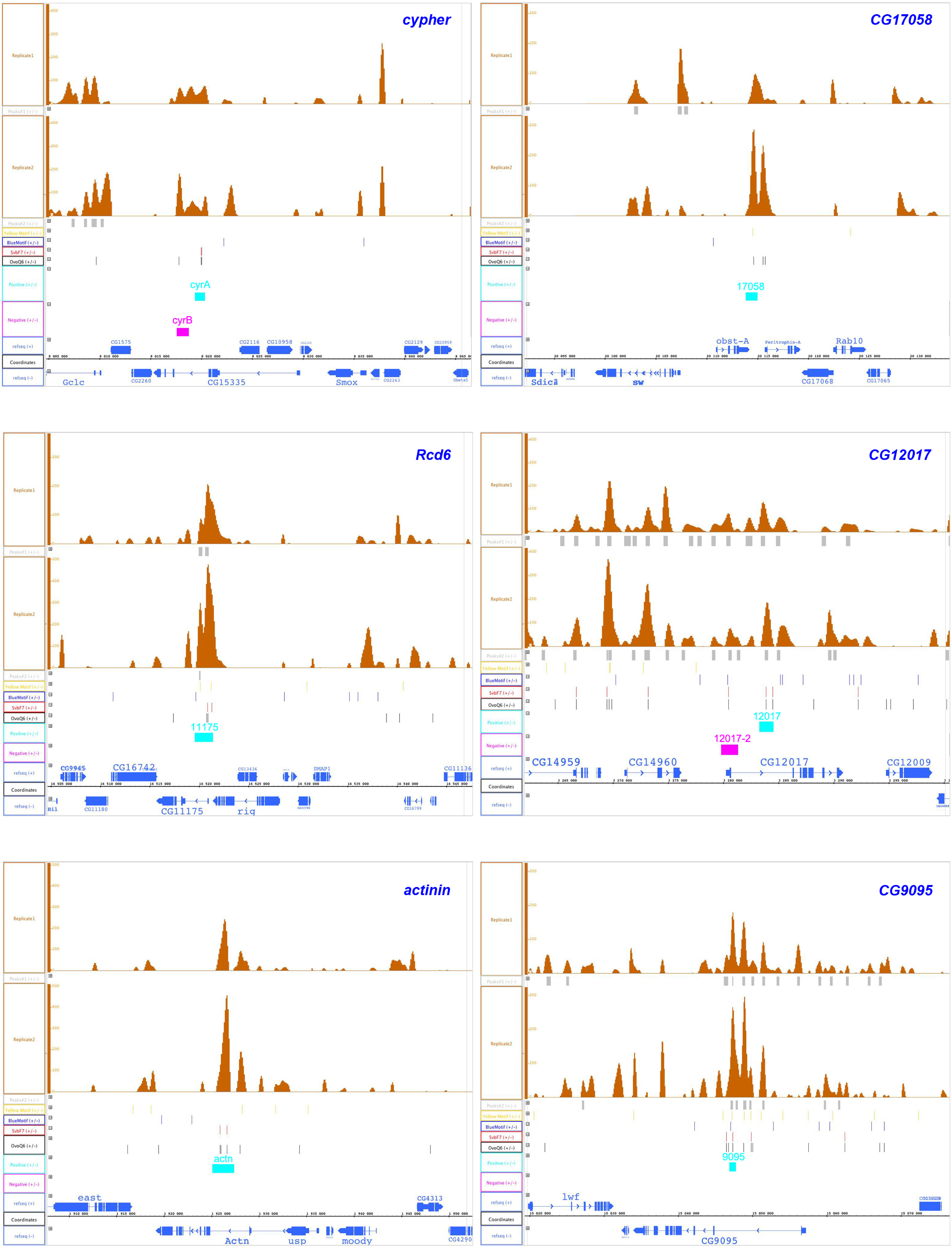

Menoret et al.; Fig S7C

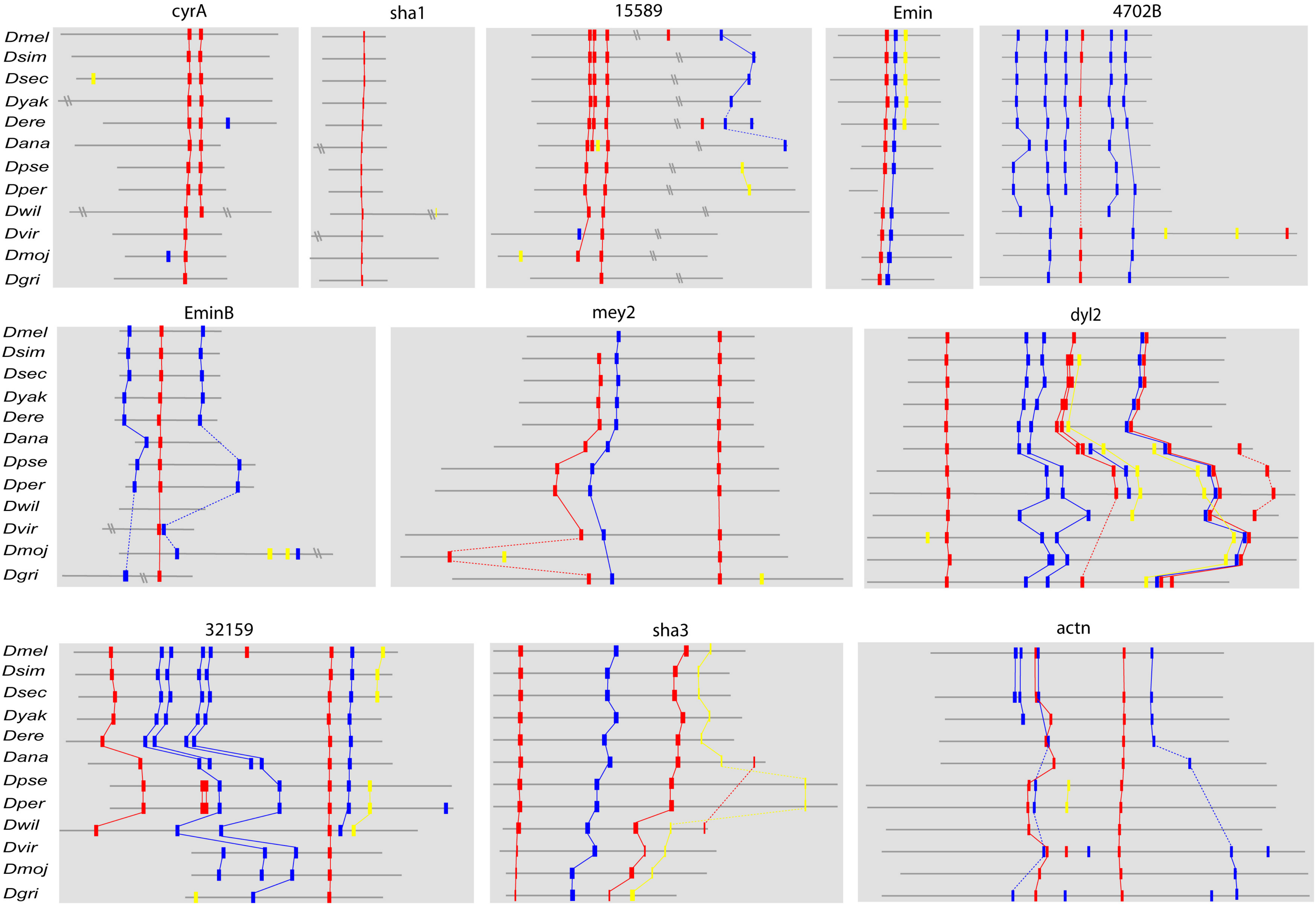

Menoret *et al.;* Figure S8A

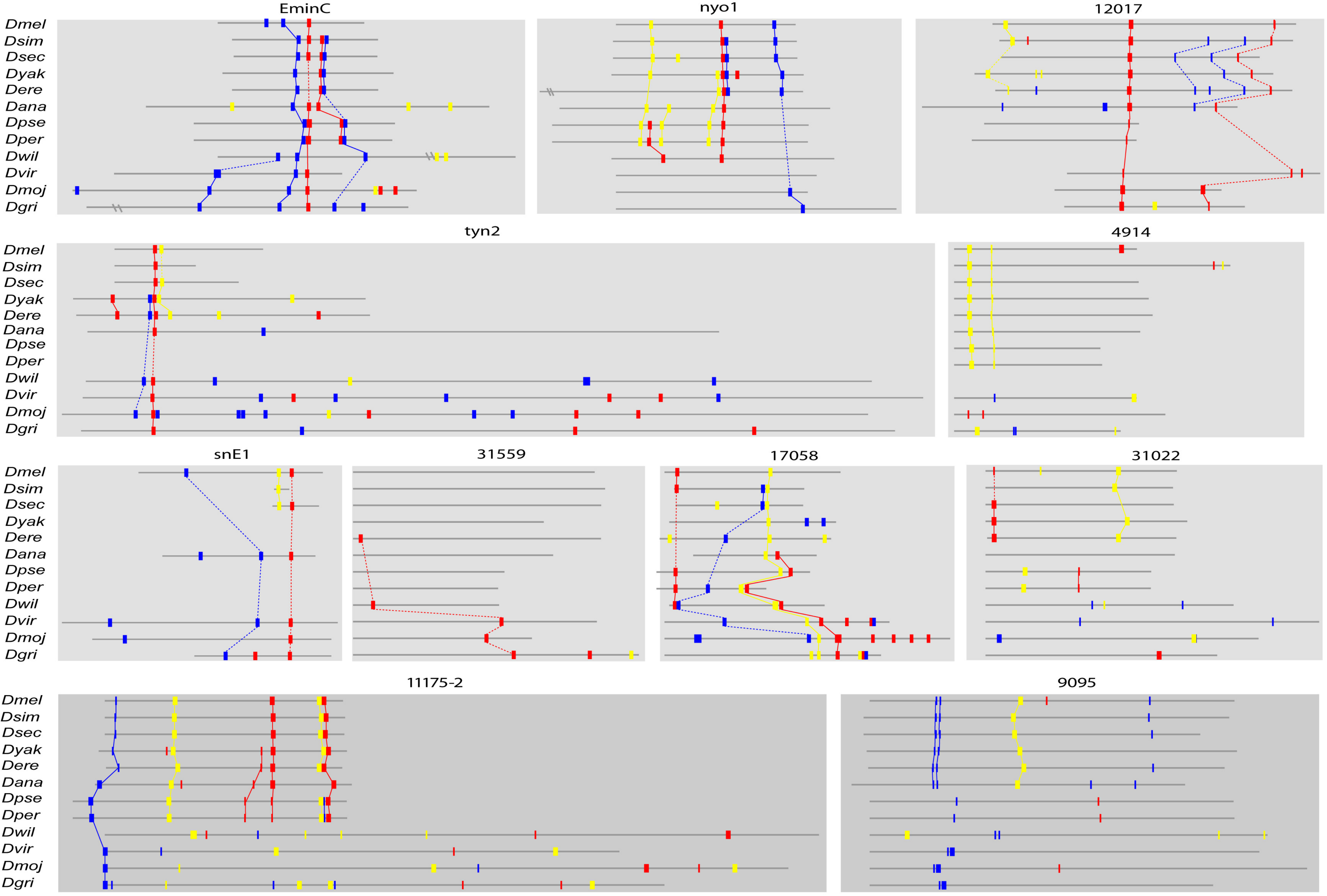

Menoret *et al.*; Figure S8B
