# Supplementary material for: Genome-wide analyses of Shavenbaby target genes reveals distinct features of enhancer organization
Source: Genome Biol. 2013 Aug 23;14(8):R86. doi: 10.1186/gb-2013-14-8-r86 (PMC4053989; doi:10.1186/gb-2013-14-8-r86)
Supplement: Additional file 2 — Legends to the supplementary figures. [file gb-2013-14-8-r86-S2.DOCX]

**Figure S1A : Identification of 21 additional Svb-downstream genes**

Genes displaying expression in subsets of epidermal were selected from the database of expression patterns, developed by the Berkeley Drosophila Genome Project (http://insitu.fruitfly.org/cgi-bin/ex/insitu.pl). mRNA expression was compared between wild type (*wt*) and *shavenbaby (svb)* mutant embryos by *in situ* hybridization. These 21 genes show a reduced expression in the absence of Svb, as documented by ventral views, with the exception of CG12814, CG14395, CG15005, CG31559 and CG31973 representing laterals views and CG15022 a dorsal view. That the expression of these genes depends on *svb* activity was further confirmed by their up-regulation following ectopic expression of Svb in the epidermis (not shown).

**Figure S1B : Epidermal genes independent of Svb**

36 genes expressed in subsets of epidermal cells showing no significant modification of their expression in *svb* mutant embryos, when compared to *wt* control. This defines a set of epidermal genes used as negative control in motif discovery approaches.

**Figure S1C : Motif prediction and CRM activity.**

**Top**: Motifs predictions in Svb downstream genes and control epidermal genes, using cisTargetX (http://med.kuleuven.be/cme-mg/lng/cisTargetX/). The predicted motifs are ranked according to their enrichment within each set compared to all *Drosophila* genes, and their evolutionary conservation. Within the set of 36 epidermal genes that are independent of Svb (left), highly ranked motifs include binding sites associated with transcription factors involved in general epidermis differentiation, such as Grh, cEBP/Vri, but no Ovo/Svb-like motifs. In the set of 39 Svb downstream genes (middle), 4 of the top 5 motifs are related to the Ovo/svb binding site, all sharing the core sequence (CnGTT or AACnG in the reverse orientation). Upon their addition in the cisTarget library of motifs (right), svbF7 and blue motifs became the first and third most enriched motifs, respectively. The use of svbF7 also increased the accuracy of enhancer prediction when compared to OvoQ6, with three additional Svb-dependent enhancers (*32159, Emin, EminB,* in cyan) detected in the top100 cisTarget predictions, and 9 negative regions (pink) no longer predicted by cis-Target (*f2, sox21b, snH5, sha-intron, f5, f4, cyrB, snP & snE4*). **Bottom:** Expression pattern of 6 additional trichome enhancers identified during initial stages of our study. These enhancers drive reporter expression in trichome cells (lacZ immuno-staining, brown), reproducing fully or partially endogenous expression of their respective genes, as assayed by *in situ* hybridization to mRNA (purple). Reporter expression was strongly reduced in *svb* mutant embryos, showing that the activity of these enhancers depends on Svb function. Tested regions were selected from different attempts of predictions, based on putative evolutionary footprinting (*EminB*), manual examination of OvoQ6-related motifs (*17058, 31559*) or an earlier version of cisTarget (version1, genome release4) for *4702B, tyn2* and *32159*. While *EminB* and *32159* become predicted by cisTargetX following the introduction of svbF7, other active enhancers do not, due to a lack of motif clustering and/or evolutionary conservation.

**Figure S1D : comparison of the predictive efficiency of various Ovo/Svb related PWMs**

**Top**: SvbF7, ovoQ6 as well as additional Ovo/Svb related PWMs (as extracted from the Fly Factor Survey database, http://pgfe.umassmed.edu/ffs/) were used with *i*-cistarget to analyse the set of 39 *svb* downstream genes. PWMs are ranked according to their enrichment score. Logo representation highlights differences in nucleotide composition and/or relative weight between PWMs. **Bottom:** Pareto plots comparing the efficiency the five Ovo/Svb-related PWMs in discriminating between the 14 functional enhancers and 25 negative regions using motifs conserved across *Drosophila* species (left) or all motifs present in *D. melanogaster* genomic regions (right). SvbF7, and to a lesser extent OvoQ6, performs better than Ovo_FlyReg, ovo_SOLEXA or ovo_SANGER that detect more false negatives (x axis) and false positives (y axis).

**Figure S2A : Architecture of cis-regulatory motifs within trichome enhancers**

Graphs plot the distance measured between all possible combinations of homotypic pairs of svbF7 and blue motifs (F7-F7 and bm-bm, resp.) and of the distance between svbF7 and either a blue (F7-bm) or a yellow motif (F7-ym). These analyses did not revealed obvious bias in the positioning of cis-regulatory motifs, as quantified by the absolute distance (bp) or relative to helical periodicity (expressed as the percentage of DNA helix rotation)

**Figure S2B : Distribution of cis-regulatory motifs associated with Svb regulated genes.**

Distribution of svbF7, blue or yellow motifs within the whole set of Svb-regulated genes (150 genes defined from microarrays) *versus* the set of control genes (100 genes from microarrays), as estimated by the number of detected motifs *per* gene. **Left panel**: The graph plots the number of evolutionarily conserved svbF7 and blue motifs detected in each set of genes. *** indicates a p-value <0,001, ** <0,01. **Right panel**: A significant enrichment for svbF7 alone, or in combination with blue (bm) or yellow motifs (ym) is detected in the set of Svb-regulated genes when compared to control genes. To avoid over-fitting, the positives sequences (CRMs) used in Fig. 3 for *de novo* motif discovery were masked prior analyses. The combination of svbF7 and blue motif exhibits higher selectivity (<5% FPR), albeit reducing sensitivity of detection. In addition, prediction with svbF7+blue or svbF7+yellow is higher (more sensitive) than with only svbF7+blue (or with only svbF7+yellow ), indicating that a subset of Svb regulated-genes are predicted by the svbF7+blue combination, whereas others are predicted using the svbF7+yellow combination.

**Figure S3 : Genes regulated by Svb as deduced from microarray profiling.**

For microarrays analysis, we focused on genes showing significant levels of expression in wild type embryos, at the temporal stage examined. From this list of 5000 genes, 150 of them displayed down-regulation in *svb* mutant and in *pri* mutant embryos. Genes are ranked according to their expression levels in *svb* mutant embryos, expressed as the percentage of wild type levels. Levels of residual expression relative to *wt* are indicated for RNA samples extracted from *pri* and *svb* mutants. Further validation of candidate target genes was performed by *in situ* hybridization in embryos mutant for *svb* (see Fig. S4)*,* or manipulated to drive ectopic *svb* expression. For each gene, the chart indicates known or putative function and protein domain, expression pattern in the epidermis and additional embryonic tissues. It also summarizes the presence of associated svbF7, blue or yellow motifs. ChIP peaks (at two developmental stages) were associated with the closest genes when located in a 5kb window upstream and downstream the transcribed region plus introns. Bona fide Svb-target genes are highlighted in green, tested genes that displayed no modifications of their expression pattern in modified *svb* genetic backgrounds are in grey.

**Figure S4 : Experimental validation of novel Svb target genes identified from microarrays.** Gene expression was assayed by *in situ* hybridization, comparing patterns observed in wild type (left panels) and *svb* mutant embryos (right panels). These 21 genes displayed a reduction in their mRNA levels in trichome cells in the absence of *svb*, while additional expression domains were unaffected, providing internal controls for specificity.

**Figure S5 : Analysis of Svb-bound regions**

**Top**: Cross-correlation between conserved svbF7 (red), blue or yellow motif instances and Svb ChIP peaks associated with either Svb regulated genes (left) or control genes (right). Plots show numbers of svbF7, blue and yellow motifs found in a 10kb window on each side of the center of peaks. **Bottom**: Histogram of p-values corresponding to cross-correlation tests between conserved svbF7 (red), blue or yellow motifs and Svb ChIP peaks, as defined from two independent ChIP-seq replicates, and their reproducibility analysis using the IDR package (https://sites.google.com/site/anshulkundaje/projects/idr).

**Figure S6 : Motif analysis of ChIP peaks associated with Svb regulated or control genes.**

Svb-bound sequences associated with Svb-regulated and control genes were subjected to *de novo* motif discovery, using the Peak Motifs computational pipeline from the Regulatory Sequence Analysis Tools package (<http://rsat.ulb.ac.be>). Enriched motifs are listed according to their rank and the corresponding logo build from *de novo* discovery is indicated. Each discovered motif was compared and aligned to known TF binding sites when showing substantial overlap. Within peaks associated with Svb regulated genes, motif 3 (tACcGTTAs) extensively matches svbF7 (ACnGTTAg) and motif 9 shows limited similarity to the blue motif.

**Figure S7 : ChiP-seq profiles of 18 Svb regulated genes**

Screen shot views from the Integrated Genome Browser (<http://www.bioviz.org/igb>, Nicol & al, Bioinformatics 2009) of ChIP-Seq signals collected in the two independent replicates. ChIP-peaks called from MACS analysis are shown under each ChIP-seq profile. Conserved svbF7 (red), OvoQ6 (black), blue and yellow motifs are drawn as vertical bars. Enhancers (positives) are shown as cyan boxes, negative regions in pink.

**Figure S8 : Evolution of the distribution of cis-regulatory motifs within trichome enhancers, across *Drosophila* species.**

Schematic representation of the distribution of svbF7 (red) blue and yellow motifs for each enhancer region, across *Drosophila* species. For motif detection, individual sequences from *D. melanogaster* and each of the orthologous regions taken from the 11 additional *Drosophila* species were processed independently, using the same threshold for the three motifs. Orthologous regions were aligned with respect to the best-conserved svbF7 site. Cis regulatory motifs that are well conserved and traceable across species are connected by full lines. Motifs for which the pattern of conservation is inferred from a parsimonious guess are connected by dashed lines. Trichome enhancers were regrouped along those showing strong (A) or more relaxed (B) conservation in the positioning of cis-regulatory motifs.
