# Supplementary material for: Genome-wide analyses of Shavenbaby target genes reveals distinct features of enhancer organization
Source: Genome Biol. 2013 Aug 23;14(8):R86. doi: 10.1186/gb-2013-14-8-r86 (PMC4053989; doi:10.1186/gb-2013-14-8-r86)
Supplement: Additional file 3 — Supplementary information (details of experimental procedure, constructs, and so on). [file gb-2013-14-8-r86-S3.DOCX]

**Supplementary informations**

**1. Genomic analyses and statistical Methods**

***De novo* motif generation**

We used the phylogeny-based *de novo* motif generation algorithm described in Rouault et al. (2010), available on the website (https://github.com/hrouault/Imogene/). The 14 positive CRMs were used as the training set for the algorithm and scanned for conserved motifs as described in (Rouault et al, 2010). The score threshold for motif generation, which sets the searched PWM information content, was varied from 7 to 13 bits in different runs of the algorithm, with a motif width set to 10 bp. In each run, the 5 highest scoring motifs were kept. This resulted in a large number of different motifs. In order to find the most discriminative ones, the 27 negative CRM were used as a negative set.

Positive and negative sets were used to evaluate the False Negative Rate (FNR) and False Positive Rate (FPR) for all motifs generated by the algorithm. For each motif, the two sets were scanned for conserved instances with a scanning threshold varied between 7 and 13 bits. For each threshold, FPR and FNR were computed as the proportion of Positive (resp. Negative) CRMs with at least one conserved instance for the motif with a score higher than the threshold. The best motifs, shown as red and blue dots, were selected based on the minimization of both FPR and FPR in a Pareto plot, as shown in Fig. 3B. These motifs were generated with a threshold of 10.1 bits and were scanned with optimal thresholds of 10.1 and 8.7 bits respectively.

**Genome-wide ranking of enhancers and genes**

In order to rank enhancers genome wide, we followed the method presented in Rouault et al, (2010). Coding sequences as well as the training set used for motif generation were masked. Conserved instances of *de novo* svbf7 and blue motifs at optimal threshold were then determined genome wide. Genomic fragment of 1Kbp were scored according to the additive Poisson score introduced in (Rouault et al., 2010) using the negative enhancers as a background set of intergenic fragments. Around each determined motif instance, the optimal scoring 1Kbp genomic fragment was defined as a putative enhancer. Each putative enhancer was associated to the nearest gene transcription start site. Each gene was attributed the highest score among its associated enhancers, or 0 if it had no associated enhancers.

**Statistical analyses**

To test for putative enrichment in a given motif between Svb-regulated and control set of genes (fig S2B), we used a Mann-Whitney U test using the transcribed region of each gene extended to 5 kb flanking sequences. A p-value was computed using the function wilcox.test from the R stats package. For motif *vs* ChIPseq cross-correlations (Fig. 6A & S5), we performed a c^2^-test to disentangle cross-correlation signals from small number fluctuations. Correlation data were binned in 500bp elements in a +/-10 kb region around the center of each ChIP peak, resulting in k=40 bins. A c^2^ was computed as the sum over the bins of the standardized counts S_i_ (O_i_ - E)^2^ / E, where O_i_ represents the observed count in bin i and E is the expected number of counts in a bin, taken to be uniform over the considered region. Finally, a p-value was computed as the probability that a c^2^ statistics with k-1 degrees of freedom takes at least the observed value.

**ChIP-seq analyses**

Sequence data was analyzed using a virtual machine image on the Bionimbus cloud (http://www.bionimbus.org/) and aligned to the *D. melanogaster* genome using Bowtie (http://bowtie-bio.sourceforge.net/index.shtml) (Langmead, 2009). Sequence density along the genome was visualized using wig files generated with SPP (Kharchenko, 2008) and sequence enrichment along the genome was defined by MACS with the following parameters: tag size=36, bandwidth=100, Pvalue=1e-5 (Zhang, 2008). files can be downloaded from the Gene Expression Omnibus public database (http://www.ncbi.nlm.nih.gov/geo/query/acc.cgi?acc=GSE48791).

ChIP peaks were subjected to motifs detection using *i-cis*targetX (http://med.kuleuven.be/lcb/i-cisTarget/) (Herrmann et al, 2012) and Peak motif (RSA tools) (http://rsat.ulb.ac.be/peak-motifs_form.cgi) (Thomas-Chollier et al. 2012).

**Genome alignments**

The alignments have been engineered by A. Caspi with the help of the Mercator and MAVID programs and were downloaded from <http://www.biostat.wisc.edu/>~cdewey/fly_CAF1/data. They were processed through a customized script to produce alignments in fasta format, mask for coding sequences (CDS) and simple repeats using the coordinates from the gff file present at <ftp://ftp.flybase.net/releases/FB2011_06/dmel_r5.38/gff>.

**References**

Hermann C, Van de Sande B, Potier D, Aerts S (2012) i-cisTarget: an integrative genomics method for the prediction of regulatory features and cis-regulatory modules. Nucleic Acids Res 2012 40(15): e114.

Kharchenko PV, Tolstorukov MY, Park PJ (2008) Design and analysis of ChIP-seq experiments for DNA-binding proteins. Nat Biotechnol 26: 1351-1359.

Langmead B, Trapnell C, Pop M, Salzberg SL (2009) Ultrafast and memory-efficient alignment of short DNA sequences to the human genome. Genome Biol 10: R25.

Rouault H, Mazouni K, Couturier L, Hakim V, Schweisguth F (2010) Genome-wide identification of cis-regulatory motifs and modules underlying gene coregulation using statistics and phylogeny. Proc Natl Acad Sci U S A 107: 14615-14620.

Thomas-Chollier M, Herrmann C, Defrance M, Sand O, Thieffry D, van Helden J (2012) RSAT peak-motifs: motif analysis in full-size ChIP-seq datasets. Nucleic Acids Res. 40(4): e31.

Thomas-Chollier M, Darbo E, Herrmann C, Defrance M, Thieffry D, van Helden J (2012) A complete workflow for the analysis of full-size ChIP-seq (and similar) data sets using peak-motifs. Nat Protoc 7(8): 1551-1568.

Zhang Y, Liu T, Meyer CA, Eeckhoute J, Johnson DS, et al. (2008) Model-based analysis of ChIP-Seq (MACS). Genome Biol 9: R137.

**2. List of transgenic constructs**

| **name** | ***gene*** | **position** | **transgene** |
| --- | --- | --- | --- |
| 11175 | *Rcd6* | chr2R 16519573 16521416 | PhiC31 |
| 12017 | *CG12017* | chr3L 3283270 3284580 | PhiC31 |
| 12017-2 | *CG12017* | chr3L 3279813 3281368 | PhiC31 |
| 12063 | *morpheyus* | chr3R 27320842 27321073 | P-element |
| 12063-2R | *morpheyus* | chr3R 27320842 27321073 | PhiC31 |
| 14395-2 | *CG14395* | chr3R 8494910 8496045 | PhiC31 |
| 1499-1 | *nyobe* | chr3R 27370295 27370415 | P-element |
| 15013-1 | *dusky like* | chr3L 4299768 4299927 | P-element |
| 15013-2 | *dusky like* | chr3L 4300420 4300715 | P-element |
| 15589 | *CG15589* | chr3R 2027050 2028050 | PhiC31 |
| 17058 | *Peritrophin-A* | chrX 20113835 20115016 | PhiC31 |
| 17058ymt | *Peritrophin-A* | chrX 20113835 20115016 | PhiC31 |
| 31022 | *PH4alphaEFB* | chr3R 26297285 26298528 | PhiC31 |
| 31559 | *CG31559* | chr3R 1977200 1978200 | PhiC31 |
| 32159 | *dsx-c73A* | chr3L 16435341 16436341 | PhiC31 |
| 32356 | *ImpE1* | chr3L 8370153 8371153 | PhiC31 |
| 4702 | *CG4702* | chr3R 7950428 7950586 | P-element |
| 4702B | *CG4702* | chr3R 7951250 7952250 | PhiC31 |
| 4914 | *CG4914* | chr3L 14670764 14672083 | PhiC31 |
| 9095 | *CG9095* | chrX 15046901 15047823 | PhiC31 |
| Actn | *actinin* | chrX 1925087 1927414 | PhiC31 |
| cyrA | *cypher* | chrX 8019397 8020397 | PhiC31 |
| cyrB | *cypher* | chrX 8017597 8018797 | PhiC31 |
| dyl1 | *dusky like* | chr3L 4303968 4304768 | PhiC31 |
| dyl2 | *dusky like* | chr3L 4298268 4299468 | PhiC31 |
| dyl2F7mtA | *dusky like* | chr3L 4298268 4299468 | PhiC31 |
| dyl2F7mtABC | *dusky like* | chr3L 4298268 4299468 | PhiC31 |
| dyl2F7mtB | *dusky like* | chr3L 4298268 4299468 | PhiC31 |
| dyl2F7mtC | *dusky like* | chr3L 4298268 4299468 | PhiC31 |
| dyl3 | *dusky like* | chr3L 4305468 4306468 | PhiC31 |
| Emin | *miniature* | chrX 11650982 11651144 | PhiC31 |
| Emin10mt | *miniature* | chrX 11650982 11651144 | PhiC31 |
| Emin11mt | *miniature* | chrX 11650982 11651144 | PhiC31 |
| Emin12mt | *miniature* | chrX 11650982 11651144 | PhiC31 |
| Emin13mt | *miniature* | chrX 11650982 11651144 | PhiC31 |
| Emin2mt | *miniature* | chrX 11650982 11651144 | PhiC31 |
| Emin3mt | *miniature* | chrX 11650982 11651144 | PhiC31 |
| Emin4mt | *miniature* | chrX 11650982 11651144 | PhiC31 |
| Emin5mt | *miniature* | chrX 11650982 11651144 | PhiC31 |
| Emin6mt | *miniature* | chrX 11650982 11651144 | PhiC31 |
| Emin7mt | *miniature* | chrX 11650982 11651144 | PhiC31 |
| Emin8mt | *miniature* | chrX 11650982 11651144 | PhiC31 |
| Emin9mt | *miniature* | chrX 11650982 11651144 | PhiC31 |
| EminA | *miniature* | chrX 11654097 11655097 | PhiC31 |
| EminAA | *miniature* | chrX 11654097 11655097 | PhiC31 |
| EminAG | *miniature* | chrX 11654097 11655097 | PhiC31 |
| EminB | *miniature* | chrX 11654097 11655097 | PhiC31 |
| Eminbmt | *miniature* | chrX 11650982 11651144 | PhiC31 |
| EminC | *miniature* | chrX 11652670 11654154 | PhiC31 |
| EminF7mt | *miniature* | chrX 11650982 11651144 | PhiC31 |
| Eminflkmt | *miniature* | chrX 11650982 11651144 | PhiC31 |
| Eminymt | *miniature* | chrX 11650982 11651144 | PhiC31 |
| f1 | *forked* | chrX 17153478 17154756 | P-element |
| f2 | *forked* | chrX 17159378 17160246 | P-element |
| f4 | *forked* | chrX 17162096 17163096 | PhiC31 |
| f5 | *forked* | chrX 17158996 17160096 | PhiC31 |
| mey2 (12063-2) | *morpheyus* | chr3R 27325605 27326605 | PhiC31 |
| Neyo | *neyo* | chr3R 25647300 25648300 | P-element |
| nyo1 | *nyobe* | chr3R 27384231 27384921 | PhiC31 |
| nyo1F7mt | *nyobe* | chr3R 27384231 27384921 | PhiC31 |
| nyo1ymt | *nyobe* | chr3R 27384231 27384921 | PhiC31 |
| nyo2 | *nyobe* | chr3R 27381479 27382574 | PhiC31 |
| nyo3 | *nyobe* | chr3R 27377275 27378274 | PhiC31 |
| sha-int | *shavenoïd* | chr2R 7216771 7220065 | P-element |
| sha1 | *shavenoid* | chr2R 7209659 7210257 | P-element |
| sha1F7mt | *shavenoid* | chr2R 7209659 7210257 | P-element |
| sha2 | *shavenoïd* | chr2R 7212709 7213256 | P-element |
| sha2-2R | *shavenoïd* | chr2R 7212709 7213256 | PhiC31 |
| sha3 | *shavenoid* | chr2R 7215630 7216294 | P-element |
| sha3bmt | *shavenoid* | chr2R 7215630 7216294 | P-element |
| sha3F7mtA | *shavenoid* | chr2R 7215630 7216294 | P-element |
| sha3F7mtAB | *shavenoid* | chr2R 7215630 7216294 | P-element |
| sha3F7mtB | *shavenoid* | chr2R 7215630 7216294 | P-element |
| sn-enh1 | *singed* | chrX 7864407 7869657 | P-element |
| snB2 | *singed* | chrX 7873257 7873915 | PhiC31 |
| snE1 | *Singed* | chrX 7869678 7870390 | PhiC31 |
| snE1 | *Singed* | chrX 7869678 7870390 | P-element |
| snE1bmt | *Singed* | chrX 7869678 7870390 | P-element |
| snE1F7mt | *Singed* | chrX 7869678 7870390 | PhiC31 |
| snE4 | *singed* | chrX 7871432 7872096 | P-element |
| snE5 | *singed* | chrX 7871996 7872659 | P-element |
| snH5 | *singed* | chrX 7868528 7868978 | P-element |
| snP | *singed* | chrX 7862910 7864103 | P-element |
| sox21b | *sox21b* | chr3L 14121641 14122852 | PhiC31 |
| tyn1 | *trynity* | chrX 86343 87613 | PhiC31 |
| tyn2 | *trynity* | chrX 77484 78384 | PhiC31 |
| tyn2F7mtA | *trynity* | chrX 77484 78384 | PhiC31 |
| tyn2F7mtAB | *trynity* | chrX 77484 78384 | PhiC31 |
| tyn2mtB | *trynity* | chrX 77484 78384 | PhiC31 |

**3. Microarray procedures**

Biotinylated cRNA targets were prepared, starting from 200 ng of total RNA, using the MessageAmp^TM^ Premier RNA Amplification Kit  (Ambion CAT# AM1792), according to the manufacturer recommendations. Following fragmentation, 6.5 μg of cRNAs were hybridized for 16 hours at 45^o^C on GeneChip® Drosophila Genome 2.0 Array interrogating over 18,500 transcripts (Affymetrix, Santa Clara, CA). The chips were washed and stained using the GeneChip® Fluidics Station 450 and scanned using the GeneChip® Scanner 3000 7G according to Affymetrix recommendations. Raw data (.CEL Intensity files) were extracted from the scanned images using the Affymetrix GeneChip® Command Console (AGCC) version 3.2. CEL files were further processed with Affymetrix Expression Console software version 1.1 to calculate probeset signal intensities using the statistics-based Affymetrix algorithms MAS-5.0 with default settings and global scaling as normalization method. The trimmed mean target intensity of each chip was arbitrarily set to 100.

files can be downloaded from the Gene Expression Omnibus public database (http://www.ncbi.nlm.nih.gov/geo/query/acc.cgi?acc=GSE48997).

The control set of genes was defined by genes showing significant expression in wild type and showing irrelevant variations in *svb* and *pri* mutants (p-value >0.8). The table below summarizes their documented embryonic pattern, expression levels in *svb* and *pri* mutant conditions (%of *wt*) and if ChIP peaks are present within a +/-5kb window.

**Control set of genes**

| *gene* | *CG Number* | *expression pattern* | *svb* | *pri* | *ChIP in 5kb* |
| --- | --- | --- | --- | --- | --- |
| *Ald* | CG7643 | not epidermal | 84,52 | 91,17 | no |
| *Atg18* | CG7986 | not epidermal | 100,61 | 98,82 | no |
| *Bap55* | CG6546 | not epidermal | 108,19 | 109,38 | yes |
| *bbx /// waw* | CG1414 | ND | 101,98 | 109,42 | yes |
| *betaggt-I* | CG3469 | ND | 110,78 | 130,95 | yes |
| *betaTub56D* | CG9277 | ep stripes | 105,75 | 105,22 | no |
| *bip2* | CG2009 | not epidermal | 146,43 | 130,23 | yes |
| *blow* | CG1363 | not epidermal | 101,79 | 104,82 | no |
| *Bre1* | CG10542 | ND | 108,66 | 134,50 | no |
| *bru-2* | CG43065 | ND | 118,64 | 112,13 | no |
| *bsf* | CG10302 | ND | 98,81 | 94,84 | no |
| *Cad87A* | CG6977 | ND | 109,50 | 112,09 | no |
| *CBP* | CG1435 | not epidermal | 102,78 | 91,76 | yes |
| *cenG1A* | CG31811 | not epidermal | 108,36 | 102,03 | no |
| *CG10365* | CG10366 | not epidermal | 100,39 | 101,34 | yes |
| *CG10731* | CG10731 | ND | 97,43 | 96,07 | yes |
| *CG11877* | CG11877 | not epidermal | 113,94 | 118,58 | no |
| *CG12006* | CG12006 | not epidermal | 114,23 | 113,87 | yes |
| *CG12164* | CG12164 | ND | 83,20 | 93,45 | no |
| *CG12375* | CG12375 | not epidermal | 103,42 | 122,64 | no |
| *CG12404* | CG12404 | ND | 104,22 | 90,36 | yes |
| *CG13284* | CG13284 | not epidermal | 100,89 | 92,66 | yes |
| *CG1371* | CG1371 | not epidermal | 115,46 | 100,11 | yes |
| *CG14229* | CG14229 | not epidermal | 105,43 | 109,87 | no |
| *CG14442* | CG14442 | ND | 111,93 | 120,36 | no |
| *CG14636* | CG14636 | ep stripes | 112,31 | 88,23 | no |
| *CG15099* | CG15099 | ND | 88,57 | 94,68 | yes |
| *CG17082* | CG17082 | ND | 91,18 | 94,01 | yes |
| *CG18549* | CG18549 | not epidermal | 98,92 | 90,20 | yes |
| *CG1965* | CG1965 | ND | 90,48 | 108,38 | yes |
| *CG2249* | CG2249 | ND | 97,42 | 83,94 | no |
| *CG2249* | CG2249 | ND | 97,42 | 83,94 | no |
| *CG2918* | CG2918 | epidermal ubiquitous | 103,01 | 88,58 | no |
| *CG31108* | CG31108 | not epidermal | 103,94 | 105,91 | no |
| *CG32164* | CG32164 | not epidermal | 98,31 | 125,93 | no |
| *CG32267* | CG32267 | ND | 95,97 | 115,07 | no |
| *CG32676* | CG32676 | ND | 90,95 | 93,01 | yes |
| *CG3305* | CG3305 | ND | 88,29 | 79,96 | yes |
| *CG3493* | CG3493 | ND | 126,41 | 109,19 | yes |
| *CG4210* | CG4210 | ND | 91,21 | 118,02 | yes |
| *CG4841* | CG4841 | ND | 110,54 | 95,91 | no |
| *CG5869* | CG5869 | ND | 89,39 | 108,39 | yes |
| *CG5931* | CG5931 | not epidermal | 123,35 | 92,97 | no |
| *CG6230* | CG6230 | ND | 95,47 | 123,11 | no |
| *CG6406* | CG6406 | not epidermal | 90,56 | 87,00 | yes |
| *CG6852* | CG6852 | ND | 111,03 | 109,18 | yes |
| *CG7028* | CG7028 | not epidermal | 101,44 | 109,71 | yes |
| *CG7852* | CG7852 | ND | 106,54 | 113,74 | no |
| *CG8090* | CG8090 | ND | 85,52 | 115,10 | yes |
| *CG8289* | CG8289 | not epidermal | 101,82 | 109,37 | no |
| *CG8878* | CG8878 | not epidermal | 103,04 | 96,89 | yes |
| *CG8928* | CG8928 | ND | 91,78 | 113,34 | no |
| *CG8931* | CG8931 | not epidermal | 110,33 | 111,09 | yes |
| *CG9293* | CG9293 | ND | 99,22 | 92,58 | yes |
| *CG9715* | CG9715 | ND | 119,34 | 97,89 | yes |
| *CG9776* | CG9776 | ND | 107,81 | 92,47 | no |
| *CG9917* | CG9917 | not epidermal | 126,61 | 102,54 | no |
| *Chd1* | CG3733 | ND | 97,28 | 94,33 | no |
| *crp* | CG7664 | not epidermal | 82,88 | 111,58 | yes |
| *dbr* | CG11371 | ND | 139,91 | 126,84 | no |
| *drl* | CG17348 | ep stripes | 115,07 | 78,43 | yes |
| *Fip1* | CG1078 | ND | 104,96 | 87,54 | no |
| *gek* | CG4012 | ND | 110,94 | 96,43 | no |
| *gp210* | CG7897 | ND | 110,61 | 106,95 | no |
| *gry* | CG17569 | ND | 97,01 | 89,65 | no |
| *hkl* | CG10473 | not epidermal | 97,76 | 103,84 | yes |
| *kis* | CG3696 | ND | 112,50 | 96,17 | yes |
| *Krn* | CG32179 | not epidermal | 102,68 | 108,51 | yes |
| *kst* | CG12008 | ep stripes | 127,31 | 103,42 | yes |
| *lack* | CG4943 | ND | 109,29 | 111,22 | no |
| *MBD-R2* | CG10042 | not epidermal | 99,57 | 106,58 | no |
| *mmy* | CG9535 | not epidermal | 95,52 | 97,92 | no |
| *mRpL33* | CG3712 | ND | 105,01 | 112,98 | yes |
| *mRpS11* | CG5184 | not epidermal | 114,70 | 108,13 | no |
| *mRpS11* | CG5184 | not epidermal | 114,70 | 108,13 | no |
| *msn* | CG16973 | ND | 114,08 | 115,00 | yes |
| *Nat1* | CG3845 | not epidermal | 108,49 | 82,47 | no |
| *RanBPM* | CG42236 | ND | 124,37 | 103,44 | no |
| *Rap2l* | CG3204 | ND | 108,16 | 107,35 | yes |
| *Rga* | CG2161 | not epidermal | 93,53 | 108,09 | yes |
| *Rgl* | CG8865 | not epidermal | 137,42 | 100,20 | yes |
| *RhoGAP1A* | CG40494 | ND | 73,46 | 113,54 | yes |
| *robo* | CG13521 | ND | 91,22 | 73,45 | no |
| *Rpb5* | CG11979 | not epidermal | 81,38 | 91,59 | no |
| *RpS27* | CG10423 | not epidermal | 106,16 | 76,13 | no |
| *RpS30* | CG15697 | ND | 115,96 | 77,01 | no |
| *RpS30* | CG15697 | not epidermal | 115,96 | 77,01 | no |
| *slik* | CG4527 | ND | 90,39 | 88,72 | yes |
| *Ssdp* | CG7187 | not epidermal | 111,89 | 87,21 | no |
| *Stlk* | CG40293 | not epidermal | 105,66 | 87,23 | no |
| *Taf1* | CG17603 | not epidermal | 107,83 | 109,83 | no |
| *tra2* | CG10128 | not epidermal | 95,99 | 79,72 | yes |
| *Trim9* | CG31721 | not epidermal | 96,29 | 91,91 | yes |
| *trr* | CG3848 | ND | 80,15 | 106,49 | yes |
| *ttk* | CG1856 | ep stripes | 97,50 | 80,48 | yes |
| *Ubp64E* | CG5486 | not epidermal | 130,11 | 101,59 | no |
| *Ufd1-like* | CG6233 | not epidermal | 112,22 | 110,15 | no |
| *ush* | CG2762 | ep stripes | 100,46 | 98,55 | yes |
| *vsg* | CG16707 | not epidermal | 113,76 | 107,51 | yes |
| *zormin* | CG33484 | ND | 130,13 | 114,51 | yes |

**
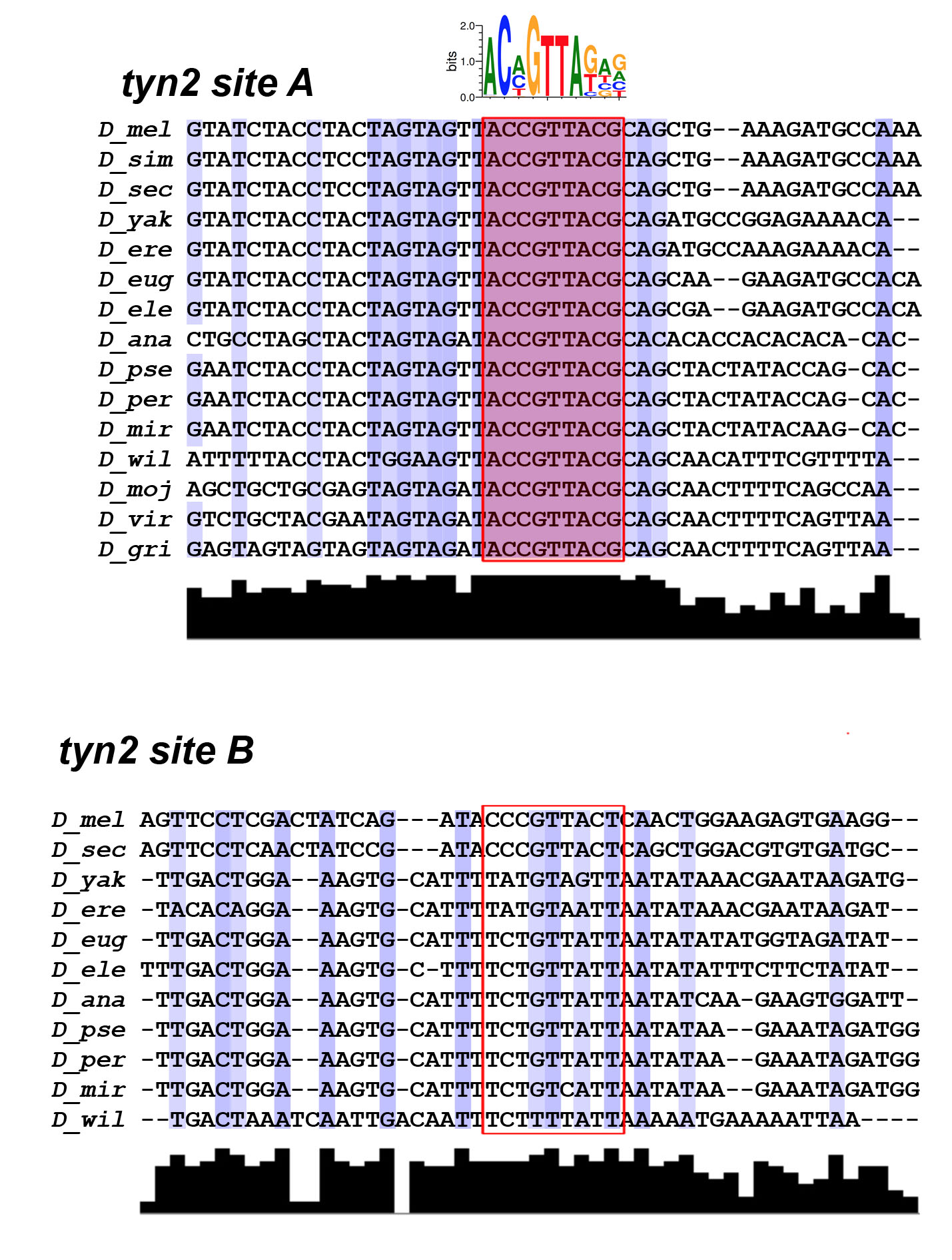
4. Evolutionary conservation of the *tyn2* enhancer**
